# Galectin-3 induces neurodevelopmental apical-basal polarity and regulates gyrification

**DOI:** 10.1126/sciadv.adt5859

**Published:** 2025-09-03

**Authors:** Luana Campos Soares, Ning Huang, Hana Bernhardova, Viviana Macarelli, Marva Chan, Lara Nikel, Sara Bandiera, Dongnan Yan, Dhanu Gupta, Elisa M. Cruz, Talia Vasaturo-Kolodner, James M. Hillis, Matthew Wood, Mootaz Salman, Zoltán Molnár, Eric O’Neill, Francis G. Szele

**Affiliations:** ^1^ Department of Physiology, Anatomy and Genetics, University of Oxford, Oxford OX1 3QX, UK.; ^2^ Kavli Institute for NanoScience Discovery, University of Oxford, Oxford, UK.; ^3^ BHF Oxford Centre of Research Excellence, University of Oxford, Oxford, UK.; ^4^ UK Dementia Research Institute, The BHF-UK DRI Centre for Vascular Dementia Research, London, UK.; ^5^ Department of Neurosurgery, Second Affiliated Hospital of Chongqing Medical University, Chongqing, China.; ^6^ Department of Oncology, University of Oxford, Oxford OX1 3QX, UK.; ^7^ Department of Bioengineering, Stanford University, Stanford, CA, USA.; ^8^ Department of Neuroscience, Yale School of Medicine, New Haven, CT, USA.; ^9^ Department of Pediatrics, University of Oxford, Oxford OX1 3QX, UK.; ^10^ Nuffield Department of Women’s and Reproductive Health, University of Oxford, Oxford, UK.; ^11^ Institute of Developmental and Regenerative Medicine, University of Oxford, Oxford, UK.; ^12^ Department of Laboratory Medicine, Karolinska Institutet, ANA Futura, Alfred Nobels Allé 8 Floor 8, SE-141 52 Huddinge, Sweden.; ^13^ Department of Neurology, Massachusetts General Hospital, Boston, MA, USA.; ^14^ Harvard Medical School, Boston, MA, USA.

## Abstract

Apical-basal polarity (ABP) establishment and maintenance is necessary for proper brain development, yet how it is controlled is unclear. Galectin-3 (Gal-3) has been previously implicated in ABP of epithelial cells, and, here, we find that it is apically expressed in human embryonic stem cells (hESCs) during neural induction. Gal-3 blockade disrupts ABP and alters the distribution of junctional proteins in hESC-derived neural rosettes and is rescued by addition of recombinant Gal-3. Transcriptomics analysis shows that blocking Gal-3 regulates expression of genes responsible for nervous system development and cell junction assembly, among others. Last, Gal-3 blockade during embryonic development in vivo reduces horizontal cell divisions, disturbs cortical layering of neural progenitors, and induces gyrification. These data uncover a regulatory mechanism for ABP in the brain and warrant caution in modulating Gal-3 during pregnancy.

## Teaser

Blocking Galectin-3 function during mice pregnancy led to de novo cortical gyrification in mouse embryos.

## INTRODUCTION

Apical-basal polarity (ABP) is an important feature in development, homeostasis, and disease, partitioning specific proteins into cellular locations to support distinct functions (*1*–*4*). Early in development, ABP of neural progenitor cells regulates morphogenesis of the neural tube. Then, ABP regulates horizontal versus vertical cell division, delamination of progeny from the apical surface of the neural tube, and basal-directed migration. In mammals, vertical cell divisions with cleavage furrows parallel to the ventricular surface generate an apical daughter cell that remains a stem cell and another daughter cell that is more differentiated. This can be a neuron, in direct neurogenesis or an intermediate progenitor that usually divides symmetrically into two neurons, in a process called indirect neurogenesis (*5*, *6*). In contrast, cell divisions in which the cleavage furrow has an oblique angle generate an apical progenitor cell and an outer radial glia (oRG) (*7*, *8*). This later cell population has been linked with gyrencephalic species and is sparse in mice (*9*). The balance of horizontal versus vertical divisions thus controls neurogenesis, making ABP essential for appropriate nervous system development.

ABP perturbation can lead to disruption in the telencephalon causing the displacement of neural progenitors in a model of hydrocephaly (*10*). Similarly, decreases in ABP-related proteins led to neuronal mispositioning in fragile X syndrome (*11*). Other disorders, such as Down’s syndrome, schizophrenia, and autism spectrum disorders, have been related to ABP impairment (*12*). Thus, ABP is essential for healthy brain growth with severe consequences when disrupted (*3*).

Many studies have begun to unravel cellular and molecular mechanisms important for ABP (*1*). Polarized exocytosis and membrane insertion of proteins is an essential aspect of ABP (*1*). Cell adhesion is strengthened via lateral junction proteins near the apical pole and is necessary for ABP function (*1*). Neuroepithelial cells in the neural tube, radial glia (RG), and in vitro neural stem cells (NSCs) derived from embryonic stem cells (ESCs) have lateral junctions and apical poles that express a variety of proteins including zonula occludens-1 (ZO-1), Par-3, N-cadherin, and γ-tubulin (*1*). The main effector of the Hippo pathway, the transcriptional coactivator Yes-associated protein (YAP), can also be recruited to the junctional complex of NSCs, where it is phosphorylated and inactivated, resulting in neuronal differentiation (*13*).

ABP can be modeled in human ESC (hESC)–derived neural rosettes, pinwheel-like cell clusters arising from apical cell constriction and basal expansion of NSCs (*1*, *14*, *15*). Thus, in addition to molecular ABP, cells can exhibit morphological ABP. For example, RG exhibit a distinctive morphological ABP consisting of apical primary cilia and long basal processes. In addition, apical versus basal positioning of cells establishes tissue level ABP (*1*, *12*). Canonical RG cell bodies are positioned apically in the ventricular zone (VZ) lining the ventricles while oRG cells are basally positioned (*16*). ABP also features in the postnatal ventricular-subventricular zone (V-SVZ). RG-like NSCs in this niche have apical primary cilia that contact the ventricles and basal processes that contact blood vessels (*17*). Ependymal cells (EC) exhibit another form of ABP in the V-SVZ. ECs have apical tight junctions (TJs) and motile cilia that are disrupted in Galectin-3 (Gal-3) knockout (KO) mice (*18*).

Gal-3 binds to β-galactoside–bearing proteins and regulates many functions critical in cancer biology including apoptosis, metastasis, and immune surveillance (*19*). Gal-3 is also important in apical trafficking and protein sorting (*20*–*22*), and its absence promotes aberrant protein distribution, which ultimately impairs epithelial morphogenesis in several models (*23*–*27*). Gal-3 also participates in ciliogenesis and spindle pole organization during cell division (*28*–*31*). In other systems, it regulates cell-cell and cell-matrix interactions (*32*–*34*) and facilitates cell growth and proliferation (*34*, *35*). During embryogenesis, Gal-3 is expressed in the trophectoderm (*36*), notochord, and embryonic macrophages (*37*). Thus, Gal-3 is expressed early during embryogenesis, but its functions in the developing brain remain unclear.

Gal-3 is expressed by reactive glia in the injured brain (*38*–*40*), is generally pro-inflammatory, and regulates angiogenesis and chemokine expression during disease (*41*, *42*). In addition to its well-known functions in disease, we discovered that it is necessary for V-SVZ neuroblast migration (*18*). Gal-3 also increases gliogenesis the postnatal V-SVZ, whereas reducing its expression reduces gliogenesis (*43*). Increasing Gal-3 expression induced astrogenesis and suppressed oligodendrogenesis, an effect dependent on BMP signaling (*43*). Gal-3 binds to the BMPR1α and increases V-SVZ BMP signaling (*43*). Gal-3 also binds to β-catenin, an important component of adherens junctions (*44*), and Gal-3 knockdown (KD) increases Wnt signaling (*45*).

Gyrification is a complex aspect of fetal cerebral cortex growth (*3*, *4*, *46*). Cerebral cortical folding includes the formation of ridges (gyri) and valleys (sulci), and it serves to increase cortical surface area, allowing this evolutionarily expanded part of the brain to occupy less volume and thus to fit into the skull. Studies point to differential proliferation of intermediate RG in the VZ and subventricular zone (SVZ) as regulating sulci and gyri (*47*, *48*). In humans, a massively expanded outer SVZ (oSVZ) is thought to give rise to the large number of neurons in our cerebral cortex and the corresponding gyrification (*49*). Several other models of cortical folding exist, but there is no consensus as to which is the most realistic (*46*, *50*, *51*).

Lower mammalian species such as mice do not exhibit cortical folds but instead have lissencephalic cortices (smooth brain). It is unclear whether lissencephalic species have mechanisms that inhibit cortical folding versus lacking mechanisms that cause gyrification (*52*). Recent work showed that YAP increases proliferation and causes mouse cortex gyrification (*53*).

Here, we found Gal-3 expression in stem cell niches of human and murine embryonic brain tissue, and we explored Gal-3 function during embryonic neurodevelopment. Gal-3 was necessary to establish ABP in human-ESC neural differentiation and the apical distribution of junctional complex proteins was lost by Gal-3 blockers in vitro. Fetal brains from female mice treated with a Gal-3 blocker unpacked TJs in the VZ, caused early delamination of NSCs and increased vertical divisions. Unexpectedly, both pharmacological blockade and genetic deletion caused sulci to appear in the developing mouse cerebral cortex.

## RESULTS

### Gal-3 is expressed apically by hESCs

**Fig. 1.**
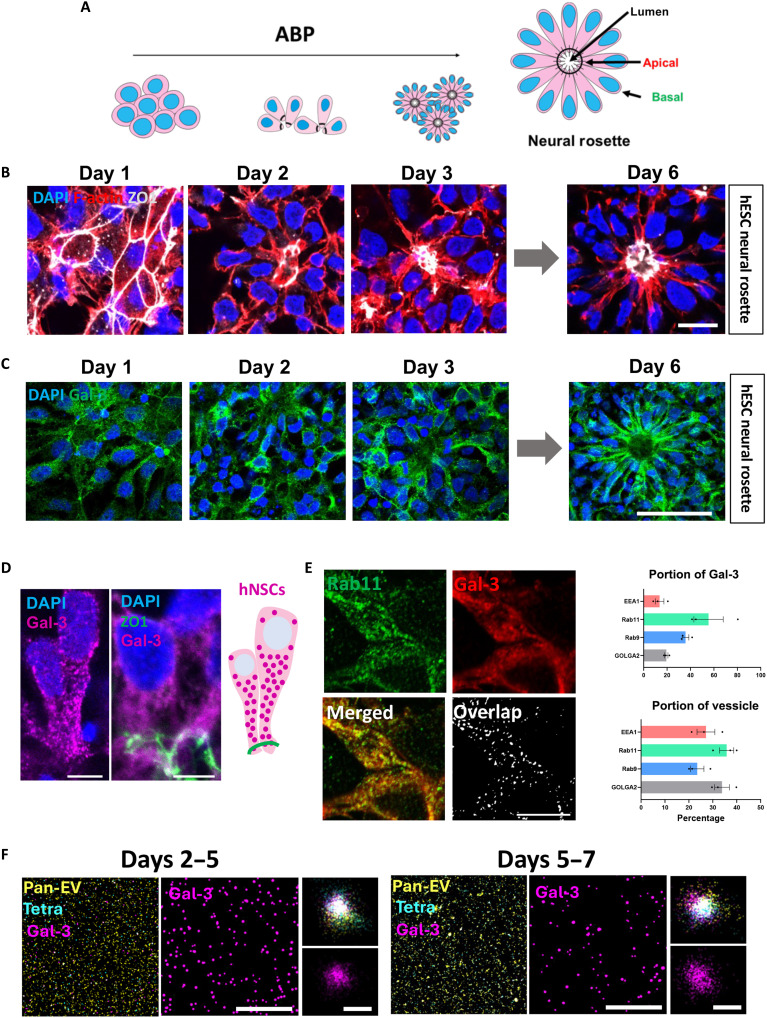
Gal-3 is apically expressed in hESC during neural rosette formation. (**A**) Schematic of neural rosettes formation in neural induction medium (NIM). Cells progressively acquired ABP until neural rosettes containing a lumen are observed at day 6. (**B**) Immunostaining of ZO-1 and F-actin proteins in hESCs over neural induction days until neural rosettes are observed at day 6. Confocal single optical section. Scale bar, 15 μm, for all panels. (**C**) Immunocytochemistry of Gal-3 in hESCs over neural induction days until full neural rosettes are formed at day 6. Confocal single optical section. Scale bar, 50 μm, applies to all panels. (**D**) Higher magnification images showing Gal-3 and ZO-1 in human NSCs (hNSCs) at day 6 of neural induction. Schematic showing the details of Gal-3’s speckled distribution and its colocalization with ZO1. Scale bar, 5 μm, in both panels. (**E**) Confocal *z*-projections of double immunocytochemistry of Gal-3 and Rab11 in day 6 hNSCs and quantification of the Manders coefficient indicating the portion of Gal-3 particles colocalized with different vesicles, and the portion of vesicles colocalized with Gal-3. Note the extensive Rab11 colocalization. Scale bars, 10 μm. (**F**) Super-resolution images of triple-positive EVs using an ONI NanoImager. As detected with immunocytochemistry, Gal-3 was colocalized with EVs (pan-EV and tetraspanin) at different time points. Higher magnification images show the details of one single triple-positive EV. Scale bars, 10 μm and 100 nm.

Neural induction medium (NIM) induces hESCs to develop into NSCs that spontaneously organize into neural rosettes (Fig. 1A). After 6 days in NIM, rosette cells exhibit ABP with constricted apical poles that contact the interior lumen and with expanded basal poles that contain nuclei (Fig. 1B). Neural rosettes are characterized by the apical localization of TJ proteins, such as ZO-1 (Fig. 1B). During rosette formation, components of the cytoskeleton, such as ZO-1 and F-actin, become apical as they reorganize expression toward the lumen (Fig. 1B). Western blotting demonstrated that hESCs express Gal-3 during neural induction with a maximum at day 2, just before the onset of rosette formation (fig. S1A). We next demonstrated Gal-3 expression in rosettes and showed that it is preferentially but not exclusively distributed toward the lumen after 6 days in NIM (Fig. 1, C and D, and fig. S1B).

Gal-3 exhibited a speckled distribution in the cytoplasm of hESC at day 6, suggesting vesicular localization (Fig. 1D). We queried whether the Gal-3 punctate expression was due to its location in specific vesicles. We found that Gal-3 was mostly colocalized with Rab11^+^ vesicles (recycling endosomes) (Fig. 1E), which matches what has been found in the literature (*21*). We found slightly less Gal-3 expressed in Rab9^+^ vesicles (late endosomes). The lowest amount of Gal-3 was found in EEA1^+^ (early endosomes) and GOLGA2^+^ (Golgi complex) (Fig. 1E and fig. S2A). We next treated cells with human recombinant Gal-3 (hrGal-3), expecting Gal-3 to be captured by early endosomes or recycling endosomes. However, there was no difference in the portion of Gal-3 or in the portion of the vesicles after treatment (fig. S2C). However, after exposure to hrGal-3, Western blots of human NSC (hNSC) lysates showed an increase in Gal-3 expression, suggesting that cells are able to capture the protein from the medium (fig. S1E).

We next used super-resolution imaging to verify whether Gal-3 is secreted via extracellular vesicles (EVs) (Fig. 1F). EVs were detected using double immunocytochemistry for pan-EV and tetraspanin. We observed Gal-3^+^ EVs in all the conditions (Fig. 1F and fig. S2, B and D); however, the percentage was higher in the medium of hESCs at days 2 to 5 (Fig. 1F and fig. S2D). Although not significant, this result suggests that at least a portion of the high levels of Gal-3 observed in cell lysates of hESCs at early days of neural induction is also being secreted via EVs and could have a nonautonomous role. Last, we detected almost four times more particles per milliliter in pluripotent cell (day 0) conditioned medium than in neural differentiation medium (fig. S2D). These in vitro data indicate that Gal-3 expression is tightly regulated during brain development and may have functional roles in ABP.

### Gal-3 l.o.f. disrupts formation and maintenance of ABP in hESCs

**Fig. 2.**
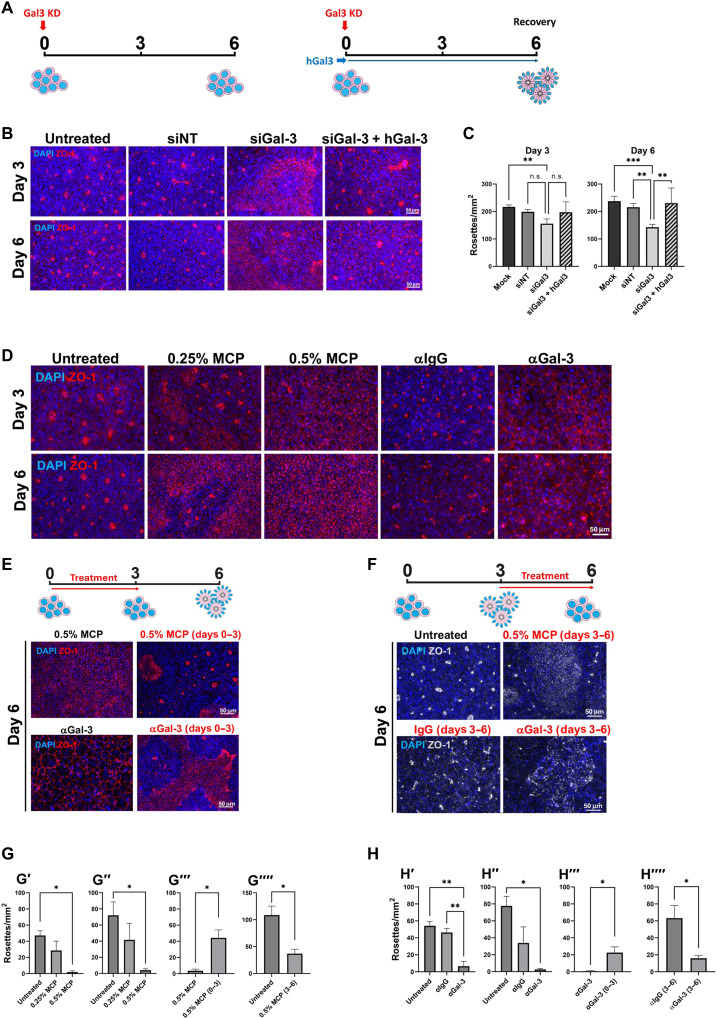
Disruption of neural rosettes by Gal-3 blockers is dynamic and reversible. (**A**) Schematics of the effects of Gal-3 KD (red arrow) and rescue with hrGal-3 (blue arrow, right). (**B**) Neural rosettes density (ZO-1 circles) at days 3 and 6. hESCs were either not transfected at day 0 (untreated control) or transfected with a nontargeting siRNA (siNT) or siGal-3. The rescue group received hrGal-3 (1 μg/ml) daily in the medium from days 0 to 6. Scale bars, 50 μm. (**C**) Quantification of (B). Graphs are means with SEM; *n* = 9 replicates (technical and biological). Two-way analysis of variance (ANOVA) followed by Tukey’s multiple comparisons test. **P < 0.01; ***P < 0.001; n.s., nonsignificant. (**D**) Neural rosettes density upon treatment with MCP or αGal-3, compared to untreated controls and control antibody αIgG, respectively. Scale bar, 50 μm. (**E**) MCP or αGal-3 treatment until day 3 (red arrow) led to a recovery in density of neural rosettes at day 6. Scale bar, 50 μm. (**F**) MCP or αGal-3 treatment from day 3 (red arrow) abolished already formed neural rosettes. Scale bars, 50 μm. (**G**) Quantification of MCP effects in (D) to (F). (G′) (day 3) and (G″) (day 6) are quantification of (D), and significance was assessed using a two-way ANOVA followed by Tukey’s multiple comparisons test. (G‴) (day 6) and (G⁗) (day 6) are quantification of (E) and (F), and significance was assessed using an unpaired *t* test. Graphs are means with SEM; *n* = 3 biological replicates; *P < 0.05. (**H**) Quantification of αGal-3 effects in (D) to (F). (H′) (day 3) and (H″) (day 6) are quantification of (D), and significance was assessed using a two-way ANOVA followed by Tukey’s multiple comparisons test. (H‴) (day 6) and (H⁗) (day 6) are quantification of (E) and (F), and significance was assessed using an unpaired *t* test. Graphs are means with SEM; *n* = 3 biological replicates; *P < 0.05; **P < 0.01.

To determine Gal-3’s role in ABP, we carried out small interfering RNA (siRNA) loss-of-function (l.o.f.) studies in rosette formation (Fig. 2, A to C; and fig. S1, C to F). ABP was assessed via the number of neural rosettes per area. ZO-1 expression in untreated rosettes is visible at low magnification as small circles of immunofluorescence (Fig. 2B and fig. S1B). The effectiveness of Gal-3 siRNA was validated with Western blotting and immunocytochemistry (fig. S1, C and D). Gal-3 KD did not affect the expression of the early neural marker and fate determinant Pax6 (fig. S1, E and F). However, it significantly disrupted neural rosette formation at day 6 after NIM (Fig. 2, B and C). Rosette formation could be rescued by adding hrGal-3 to the medium for 6 days (Fig. 2, B and C). These data indicate that Gal-3 specifically regulates ABP rather than the acquisition of neural fate.

We next confirmed these results with modified citrus pectin (MCP), a nutraceutical-derived Gal-3 inhibitor (*54*–*56*). The number of neural rosettes per square millimeter was significantly decreased at 3 and 6 days by MCP (Fig. 2, D, G′, and G″). MCP treatment led to a concentration-dependent decline in neural rosette numbers, where 0.25% MCP partially blocked and 0.5% MCP completely blocked neural rosette formation at both time points (Fig. 2D, G′ and G″). Blocking antibodies (αGal-3) were previously used to show Gal-3 is necessary for neuroblast migration (*18*). Here, rosette formation was significantly reduced, compared to the α immunoglobulin G (αIgG) control after 3 days of exposure to αGal-3 (Fig. 2, D, H′, and H″).

The loss of rosettes observed after 3 days of MCP treatment was significantly reversed when MCP was removed between days 3 and 6. This suggests that ABP is dynamic and requires continuous Gal-3 to be maintained (Fig. 2, E and G‴). Rosettes were also found at day 6 when αGal-3 was only given for 3 days compared with being given throughout (Fig. 2, E and H‴).

We next asked whether Gal-3 blockade could disrupt already generated rosettes. MCP (0.5%) included in the medium between days 3 and 6 significantly disrupted rosette formation (Fig. 2, F and G⁗). Rosettes already formed between days 0 and 3 were also disrupted by αGal-3 in the medium between days 3 and 6 (Fig. 2, F and H⁗). Neither MCP nor αGal-3 treatments changed activated cleaved caspase-3 expression, suggesting that apoptosis was not the cause of reduced rosette density (fig. S1, G and H).

Thus, l.o.f. experiments using all three Gal-3 inhibitors blocked rosette formation as established by loss of apical ZO-1. These data provide strong evidence that Gal-3 has an important role in promoting and maintenance of ABP formation during hESC rosette formation.

### Gal-3 blockade inhibits organization of apical junctions

**Fig. 3.**
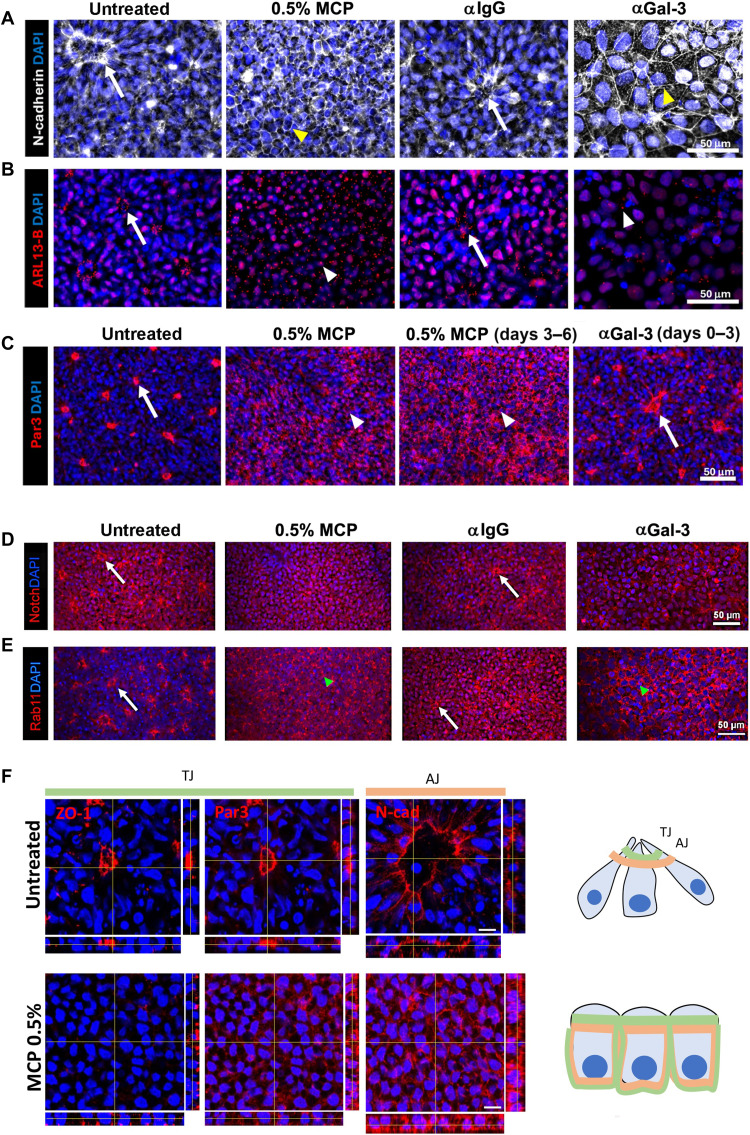
Gal-3 regulates distribution of junctional proteins. (**A**) Apical distribution of N-cadherin observed via immunocytochemistry at day 6 is disrupted upon treatment with MCP and αGal-3 since day 0. Arrows indicate apical region of fully formed neural rosettes. Arrowheads indicate redistribution of N-cadherin. Scale bar, 50 μm. (**B**) Apical distribution of Arl13-B observed via immunocytochemistry at day 6 is disrupted upon treatment with MCP and αGal-3 since day 0. Arrows indicate apical region of neural rosettes. Arrowheads indicate redistribution of Arl13-B. Scale bar, 50 μm. (**C**) Immunocytochemistry for Par3 reveals that its apical localization is disrupted upon treatment with MCP from days 0 to 6 or from days 3 to 6. In contrast, stopping the treatment with αGal-3 at day 3 allowed recovery of apical localization of Par3 at day 6. Arrows indicate apical region of fully formed neural rosettes. Arrowheads indicate redistribution of Par3. Scale bar, 50 μm. (**D**) Immunocytochemistry for Notch shows apical localization in controls and loss of this pattern after MCP and αGal-3 (compared with αIgG control). Scale bar, 50 μm. (**E**) Immunocytochemistry for Rab11 shows apical localization in controls and loss of this pattern after MCP and αGal-3 (compared with αIgG control). Scale bar, 50 μm. (**F**) Orthogonal views of tight and adherens junction proteins ZO-1, Par3, and N-cadherin in untreated and 0.5% MCP-exposed hESCs at day 6 of neural induction. Note that their expression is quite specifically localized to the apical poles of rosettes. After rosette disruption by the Gal-3 blocker MCP, these proteins moved toward the basolateral domain within the cells. Schematics on the right show the redistribution of TJ and AJ proteins after Gal-3 block. TJ, tight junctions; AJ, adherens junctions. Scale bars, 10 μm.

Gal-3 participates in the delivery of proteins to the apical pole via recycling endosomes in epithelial tissue (*20*, *24*, *25*, *28*). Thus, we queried whether Gal-3 effects on apical polarization were limited to ZO-1 or were more general. Both 0.5% MCP and αGal-3 added for 6 days to the NIM of ESCs caused loss of apical polarization of N-cadherin, ARL13-B, Par3, Notch, and Rab11 (Fig. 3, A to E). During normal rosette formation, the ciliary protein ARL13-B was localized in discreet apical puncta, which dispersed in a seemingly random distribution upon Gal-3 blockade (Fig. 3B). Notch expression was lost from its apical localization and moved into the nucleus after MCP exposure (Fig. 3D). We also found that MCP added to the medium from days 4 to 6 could disrupt already formed Par3^+^ rosettes (Fig. 3C). Treatment with αGal-3 from days 0 to 3 only, resulted in Par3^+^ rosettes reappearing by day 6 (Fig. 3C).

Orthogonal views of higher magnification confocal images revealed the polarized distribution of tight and adherens junctions in neural rosettes at day 6. This was evidenced by the presence of bigger or smaller discs of junctions surrounded by 4′,6-diamidino-2-phenylindole (DAPI)^+^ nuclei (Fig. 3F). Upon MCP treatment, this neural epithelium lost its polarity at the same time that junctional proteins moved to the basolateral domain (Fig. 3F). Among the proteins studied, N-cadherin was the most affected by MCP. These experiments showed that loss of Gal-3 function caused redistribution of all apical markers tested and was not restricted to ZO-1.

Yap is implicated in ABP (*57*, *58*) and is an important component of the junctional complex in ECs (*59*). Yap localization at TJs is also important for VZ integrity and correct delamination of progenitors (*60*). Here, hESC-derived fully formed rosettes expressed YAP at the apical pole in TJs where it colocalized with ZO-1 (fig. S3A). Apical polarization of YAP in rosettes was lost upon exposure to αGal-3 compared to αIgG controls, with most of the YAP found in the nucleus (fig. S3A). MCP treatment also promoted a shift of YAP localization from the TJ to the nucleus (fig. S3, B and C). Quantification of YAP and ZO-1 colocalization showed a significant loss upon MCP exposure, whereas YAP and DAPI colocalization increased (fig. S3D). Together, these data indicate that Gal-3 is important for the correct distribution of several apical proteins and for the recruitment of YAP to the TJs (fig. S3D).

### RNA-seq reveals dysregulation in neurodevelopmental pathways upon MCP treatment

**Fig. 4.**
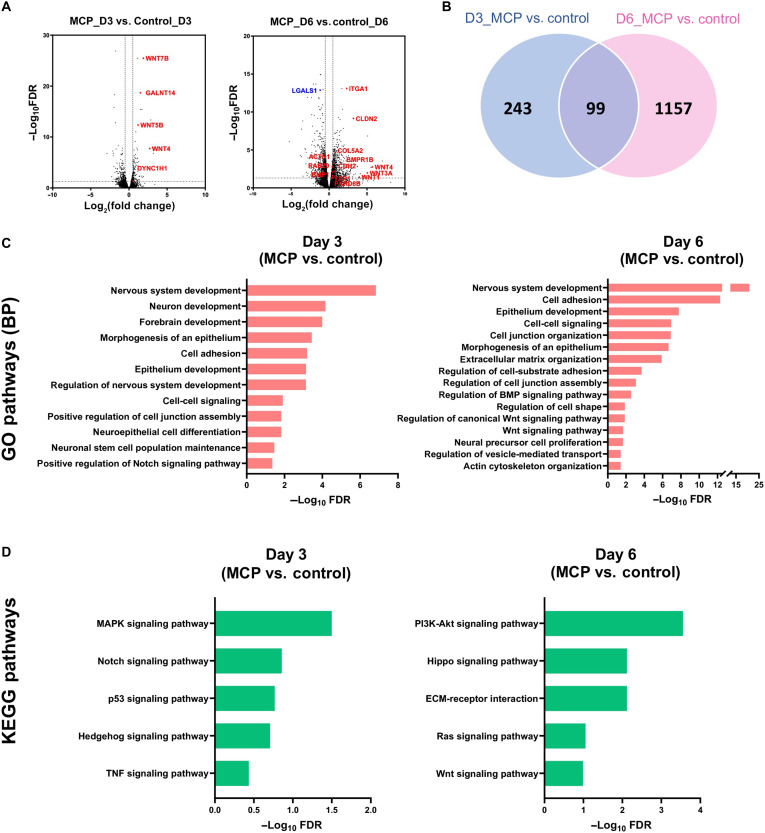
RNA-seq reveals developmental genes regulated by Gal-3. (**A**) Volcano plot of genes differentially expressed (FDR < 0.05) in MCP-treated versus control hNSCs at days 3 and 6 of neural induction. A subset of genes significantly up-regulated [Log_2_(fold change) > 0.5] is shown. (**B**) Venn diagram representing the number of genes differentially expressed in MCP versus control groups and the overlap between different days. (**C**) Gene Ontology (GO)–biological processes enrichment analysis of genes up-regulated in MCP versus control group at days 3 and 6. (**D**) Kyoto Encyclopedia of Genes and Genomes (KEGG) pathways enrichment analysis of genes up-regulated and down-regulated in MCP versus control group at days 3 and 6. MAPK, mitogen-activated protein kinase; TNF, tumor necrosis factor; PI3K, phosphatidylinositol 3-kinase.

Multiple mechanisms may participate in Gal-3’s regulation of ABP. Therefore, we carried out bulk RNA sequencing (RNA-seq) on control and MCP-treated hNSCs at days 3 and 6 of neural induction. First, principal components analysis of gene expression values fragments per kilobase million (FPKM) confirmed good segregation between the groups (fig. S4A). We then identified genes that were up- or down-regulated in MCP-treated hESCs at days 3 and 6, using stringent conditions [false discovery rate (FDR) < 0.05; |log_2_(fold change)| > 0.5] (Fig. 4A and table S2). At day 3, there were 342 genes differentially expressed in MCP-treated cells, of which 126 were up- and 216 were down-regulated (fig. S4B). This number was higher at day 6 where 1258 genes were differentially expressed (655 up and 603 down) (fig. S4B). Most genes where uniquely enriched in day 3 or day 6 MCP groups, with an overlap of 99 differentially expressed genes between the two time points (Fig. 4B). We found that several genes involved in Wnt signaling were overexpressed (Fig. 4A). Gene Ontology (GO) analysis showed that at day 3, most dysregulated biological pathways (BPs) upon MCP treatment were related to nervous system development and epithelium morphogenesis (Fig. 4C and table S3). At day 6, besides neurodevelopmental pathways, altered gene expression was also in pathways of cell junction assembly, cytoskeleton remodeling, and extracellular matrix (ECM) organization (Fig. 4C), which was supported by the cellular components analysis (fig. S4C). For example, *CDH2* was up-regulated and *CDH10* down-regulated at day 3 (table S2). *CDH2* and *CDH9* were up-regulated, while *CDH3* and *CDH17* were down-regulated at day 6. At this time point, we also found changes in expression of integrin receptors, for example, up-regulation of alpha 1 subunit of integrin receptors (*ITGA1*) and *ITGA2* and down-regulation of *ITGA4* (table S2). Pathways regulating vesicle-mediated transport were also perturbed at day 6 (table S2).

We also questioned the Kyoto Encyclopedia of Genes and Genomes (KEGG) database for dysregulated biological functions upon MCP treatment (Fig. 4D and table S4). With this analysis, we found dysregulation of Notch and Wnt signaling at day 3, as well as ECM-receptor interaction at day 6. We additionally found the Hippo pathway being dysregulated at day 6 (Fig. 4D). Up-regulation of Notch was also observed with GO analysis, including its ligands Delta-like canonical Notch ligand 1 (*DLL1*) and Jagged1 (*JAG1*) at day 3 (Fig. 4C and table S2). In contrast, at day 6, we found dysregulation of Wnt and BMP signaling pathways (Fig. 4C).

As expected, comparison between control at day 6 versus day 3 and MCP at day 6 versus day 3 showed a high overlap of differentially expressed genes, indicating that time of differentiation affects both groups similarly (fig. S4D). GO analysis showed similar enrichment in pathways regulating neural differentiation in both groups (fig. S4E).

### Human and murine embryonic brain stem cell niches express Gal-3

**Fig. 5.**
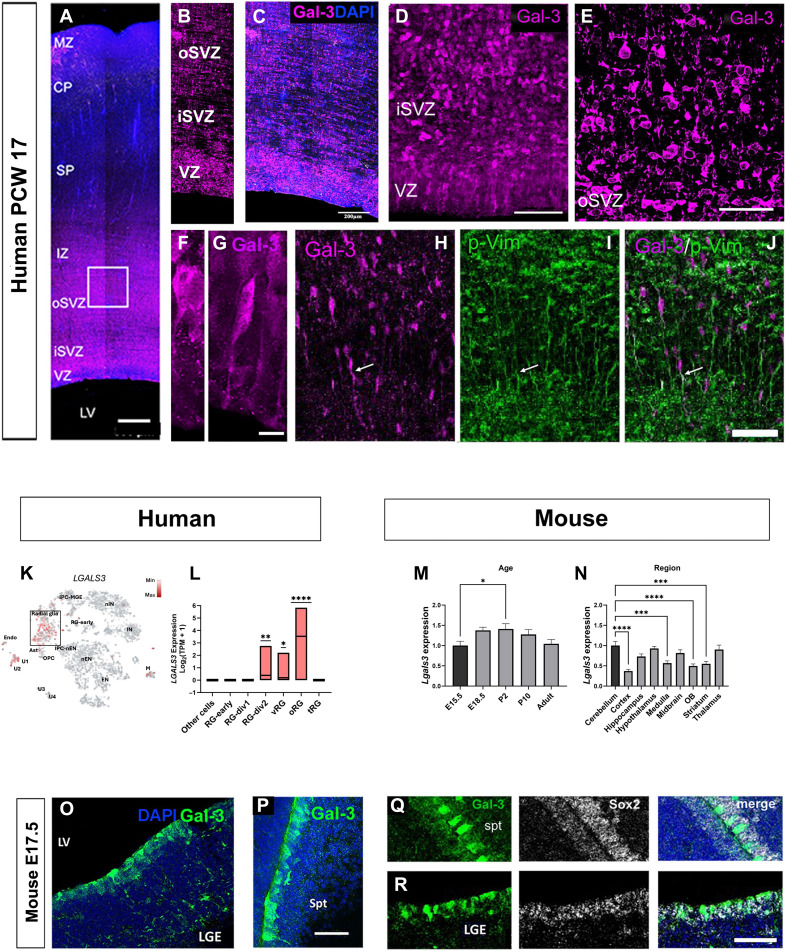
Gal-3 is expressed in human and murine embryonic NSC. (**A** to **J**) Gal-3 expression was analyzed via immunohistochemistry in coronal sections of a postconception week (PCW) 17 human embryonic brain. MZ, marginal zone; CP, cortical plate; SP, subplate; IZ, intermediate zone; oSVZ, outer subventricular zone; iSVZ, inner subventricular zone; VZ, ventricular zone; LV, lateral ventricle. (A) Robust Gal-3 expression was observed in the VZ, iSVZ, oSVZ, and IZ layers. Scale bar, 500 μm. (B and C) Details of Gal-3 immunohistochemistry in the VZ, iSVZ, and oSVZ. Scale bar, 200 μm. (D) Gal-3 expression in the VZ and iSVZ, confocal *z*-stack projection. Scale bar, 100 μm. (E) Gal-3 expression in the oSVZ, single optical section. Scale bar, 20 μm. (F and G) Higher magnification showing Gal-3–positive VZ cells with ABP, with apical process evident, and RG-like morphology. Scale bar, 10 μm. (H to J) The oSVZ showing Gal-3–positive and p-Vimentin (p-Vim)–positive RG fibers (arrow) and migrating neuroblasts. Scale bar, 50 μm. (**K**) tSNE plot showing *LGALS3* gene expression from scRNA-seq in radial progenitor populations (oRG, vRG, and RG-div2) across stages of human embryonic cortical neurogenesis. Generated using UCSC Cell Browser (*61*). Cell type clusters are annotated. Clusters RG-div1, RG-div2, oRG, vRG and tRG are boxed. PC-MGE, intermediate progenitors of medial ganglionic eminence; IPC-nEN, intermediate progenitors of newborn excitatory neurons; nIN, newborn interneurons; IN, interneurons; nEN, newborn excitatory neurons; EN, excitatory neurons; M, microglia; Ast, astrocytes; OPC, oligodendrocyte progenitors; Endo, endothelial cells; RG-early, Early radial glial cells; RG-div1 and 2, dividing radial glial cells cluster 1 and 2; oRG, outer radial glial cells; tRG, truncated radial glial cells. (**L**) Human *LGALS3* enrichment measure in transcript per million (TPM) + 1. Graph bar is minimum to maximum, and line represents mean. Wilcoxon’s rank sum test. *P < 0.05; **P < 0.01; ****P < 0.0001. (**M** and **N**) PCR showing murine *Lgals3* expression in different ages (M) and across regions (N). Fold change values were calculated relative to left-most columns. Graphs show mean with SEM. *N* = 3 for each region except for cerebellum P10 (*N* = 2) and midbrain E18 (*N* = 2) and midbrain P2. Ordinary one-way ANOVA followed by Dunnett’s multiple comparison test was used for statistical analysis. *P ≤ 0.05, ***P ≤ 0.001, ****P < 0.0001. (**O** and **P**) Gal-3 expression in E17.5 mouse lateral ganglionic eminence (LGE) (O) and septum (Spt) (P). LV, lateral ventricle. Scale bar, 50 μm. (**Q** and **R**) Gal-3 is expressed in Sox2^+^ cells lining the lateral ventricles. Scale bar, 50 μm.

Because hESC expressed Gal-3 in vitro, we hypothesized that it would also be expressed in embryonic NSCs in vivo (*18*, *43*). Gal-3 was expressed in the VZ, the inner SVZ (iSVZ) and oSVZ and in the intermediate zone (IZ) of postconception week (PCW) 17 human brain (Fig. 5, A to C). Higher magnification revealed Gal-3^+^ cells lining the LV as well as Gal-3^+^ cells in the iSVZ and oSVZ (Fig. 5, D to H). The morphology and position of Gal-3^+^ cells suggested that they were ventricular RG (vRG) (Fig. 5, F to H), oRG, or neuroblasts. Some Gal-3^+^ cells in the developing human forebrain expressed the RG marker p-Vimentin (Fig. 5, I and J). We did not detect Gal-3 immunoreactivity in the human subpallium. Gal-3 immunofluorescence was also found in human brain in the cortical sling (fig. S5D). These results indicate the presence of Gal-3 in NSC in human embryonic brains. They are commensurate with human single-cell RNA sequencing (scRNA-seq) studies showing *LGALS3* transcripts (Gal-3 gene) in multiple subtypes of embryonic RG (Fig. 5, K and L) (*61*).

Others have shown low, but detectable, levels of *Lgals3* in developing mouse RG with scRNA-seq (*62*, *63*). We detected *Lgals3* with reverse transcription quantitative polymerase chain reaction (RT-qPCR) in the mouse brain at embryonic day 15.5 (E15.5), E18.5, postnatal day 2 (P2), P10, and adulthood (Fig. 5M). All areas examined (fig. S5A) expressed detectable *Lgals3* transcripts with the cerebral cortex expressing the lowest amount (Fig. 5N). Using immunohistochemistry, we found robust Gal-3 expression in the VZ of the lateral ganglionic eminence (LGE) (Fig. 5O) and septum (Fig. 5P) of E17.5 mice. Gal-3 immunofluorescence in cell bodies lining the ventricles suggested that they were RG. The highest Gal-3 expression was apical, in cell bodies, whereas basal processes contained little to no detectable Gal-3 (Fig. 5, Q and R). Gal-3 expression also colocalized with Sox-2 (which is expressed by RG stem cells) in LGE and septal cells lining the lateral ventricle (Fig. 5, Q and R). Gal-3 also colocalized with ZO-1 (fig. S5B). Gal-3 immunofluorescence was not detected in the mouse E17.5 pallium although we detected it there with RT-PCR. As anticipated, Gal-3 expression was detected in some Iba1^+^ microglial cells in the embryonic mouse brain especially in the cortical sling, which includes the developing corpus callosum and septum (fig. S5, C and E).

### Gal-3 inhibition leads to sulcus formation

**Fig. 6.**
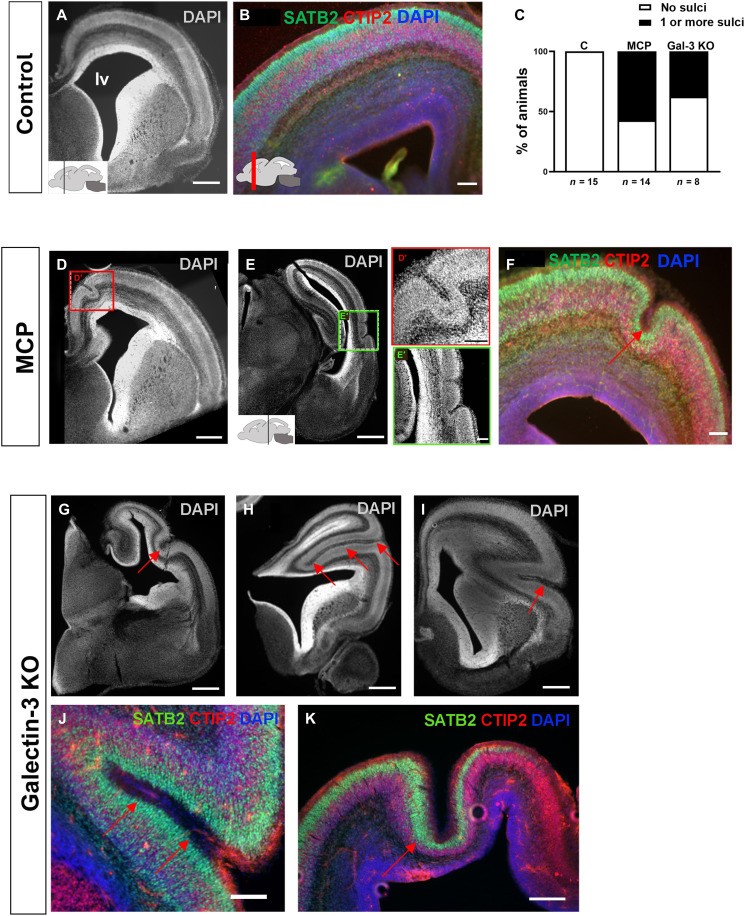
Gal-3 inhibition leads to sulci formation in E17.5 mice embryos. (**A**) DAPI staining of control coronal hemi-sections at an anterior level. Note the smoothness of the cortical surface. lv, lateral ventricle. Scale bar, 500 μm. (**B**) Control cerebral cortex at an anterior level with immunohistochemistry for Satb2 and Ctip2. Scale bar, 100 μm. (**C**) Quantification of the percentage of embryos displaying sulci in all litters studied (two litters per group). Quantification showed that controls had no sulci. (**D**) Anterior level coronal hemi-section from an embryo exposed to MCP. DAPI staining of MCP-treated embryos at similar anterior posterior level as (A) showing representative sulcus (red box). Box shown at higher magnification in (D′). Scale bar, 500 μm. (D′) 100 μm. (**E**) Posterior level coronal hemisection showing two adjacent sulci in an MCP-treated embryo. Green box shown at higher magnification in (E′). Scale bar, 500 μm. (E′) 100 μm. (**F**) Satb2 and Ctip2 immunohistochemistry with a typical sulcus (red arrow). Scale bar, 100 μm. (**G**) A Gal-3^−/−^ embryo with a typical sulcus. Scale bar, 100 μm. (**H**) Gal-3^−/−^ embryo with a deep sulcus (red arrows). Scale bar, 100 μm. (**I**) A Gal-3^−/−^ embryo with an intermediate depth sulcus (red arrow). Scale bar, 100 μm. (**J**) Satb2 and Ctip2 immunohistochemistry of the sulcus shown in (J). Scale bar, 400 μm. (**K**) Gal-3^−/−^ embryo sulcus (red arrow) stained for Satb2 and Ctip2. Note that it almost reached the VZ. Scale bar, 400 μm.

Our findings suggested that Gal-3 l.o.f. may also disturb neurodevelopment in vivo during embryogenesis. MCP is known to affect the central nervous system upon systemic injection (*55*, *64*), and we administered it to pregnant mice in their drinking water and analyzed brains of embryos at E17.5. There was no difference in water consumption or weight gain between control and MCP-treated females (fig. S6A). Control embryos had smooth cerebral cortices as expected since mice are lissencephalic (Fig. 6, A and B; and fig. S6, B and C). MCP-treated brains, in contrast, had sulci in the cerebral cortex of 57.1% of the embryos (Fig. 6, C to F; and fig. S6, B and C). This was seen across all anterior-posterior and mediolateral regions of the cerebral cortex (Fig. 6, D to F, and fig. S6C). Most embryos administered MCP had one sulcus, but two embryos had more than one (Fig. 6E). The sulci were measured using sulcus depth (SD) and local gyrification index (LGI), adapted from (*65*) (fig. S6, D and E). A higher SD indicates deeper folding, while a higher LGI signifies narrower and deeper folding. Foldings were classified as sulci if the SD value exceeded 100 μm and the LGI value exceeded 0.5 (fig. S6, D and E). The cortical layer markers Satb2 and Ctip2 exhibited proper orientation, indicating that these folds reflected cortical folds and not tissue damage (Fig. 6, B and F, and fig. S6C). We then measured the gyrification index (GI), which is the ratio between the detailed outline of the pial surface and outer contour of the cortex (fig. S6F). Higher GI values indicate more brain folding. There was a 9.2% increase in the GI value in the MCP group when compared to the control (fig. S6F).

To confirm these results, we examined two litters of Gal-3^−/−^ mice (Gal-3 KO) at E17.5 of gestation. Gross anatomical examination of brain sections revealed the unexpected result that 37.5% mice had sulci in the cerebral cortex similar to the ones that we observed after MCP (Fig. 6, C and G to K). The depth of the sulci varied from somewhat shallow (Fig. 6G) to quite deep (Fig. 6, H and I), which can be verified with the SD and LGI values (fig. S6, D and E). The cortical layer markers Satb2 and Ctip2 immunofluorescence appeared to be normal and were expressed in and around the sulci (Fig. 6, J and K). There was a 19.5% increase in the GI value in Gal-3 KO compared to control brains (fig. S6F). In addition to the cortical sulci, we observed several examples of indentations along the walls of the lateral ventricles (fig. S6G). Quantification of these VZ notches shows that there were significantly more in the KO compared to control mice (fig. S6H).

### Gal-3 blockade disrupts ABP and cell division in forebrain embryogenesis in vivo

**Fig. 7.**
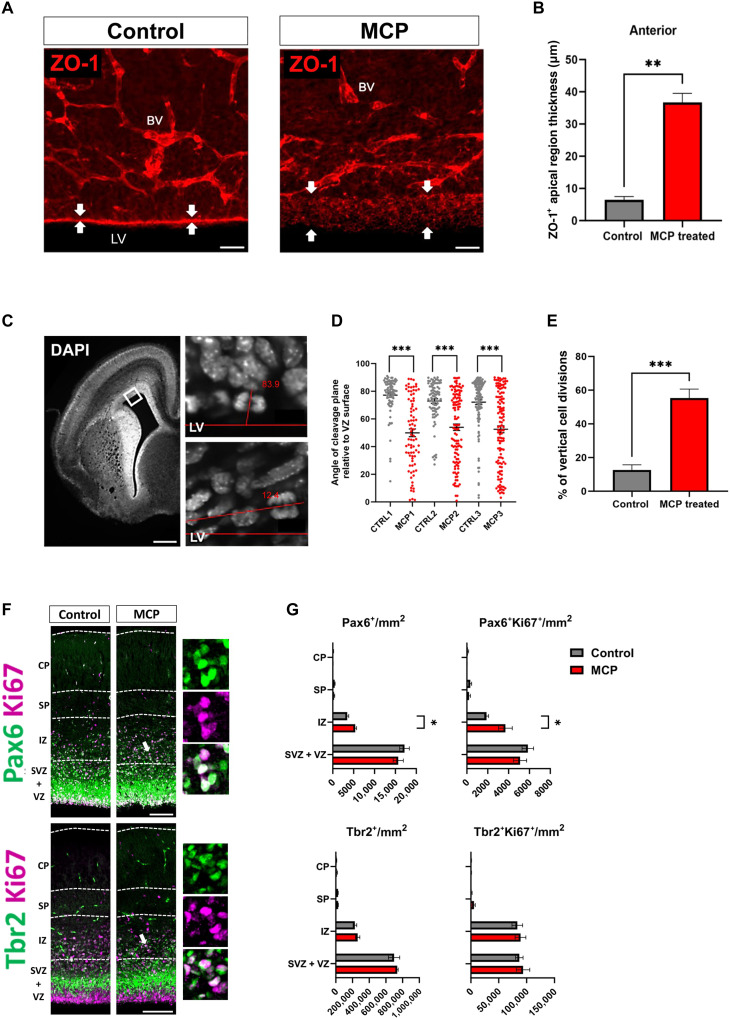
Gal-3 inhibition in the developing cortex: disrupted ABP, increased vertical cell division, and delamination from apical surface. (**A**) Representative image showing ZO1 immunohistochemistry in the pallial VZ lining the lateral ventricle (LV). Anterior cerebral cortex, coronally sectioned. Arrows indicate the thickness of the apical VZ region, which is much expanded after MCP treatment. Note also that blood vessels (BV) are ZO1^+^. Scale bars, 50 μm. (**B**) Quantification of thickness of apical region of control and MCP-treated E17.5 mice embryos in anterior sections. Graphs are represented as means with SEM; *n* = 6. Mann-Whitney test; **P < 0.001. (**C**) Representative image of DAPI staining of mouse E17.5 brain sections. Square on the left image shows enlarged area on the right images. The red lines show the angle between the cortex-lateral ventricle border and the cleavage plane of neural progenitors in anaphase. Scale bar, 350 μm. (**D**) Distributions of cleavage plane angles in separate control and MCP-treated E17.5 embryos. Graphs are represented as means with SEM; *n* = 3. Mann-Whitney test; ***P < 0.001. (**E**) Quantification of percentage of vertical cell divisions in control and MCP-treated embryos. Vertical cell divisions were classified as anaphases with a cleavage plane angle of less than 60°. Graphs are represented as means with SEM; *n* = 3. Mann-Whitney test; ***P < 0.001. (**F**) Representative images of Pax6^+^Ki67^+^ immunohistochemistry and Tbr2^+^Ki67^+^ immunohistochemistry. Images are from anterior cortex in control and MCP-treated E17.5 embryos. Arrows point toward example of PAX6^+^Ki67^+^ and Tbr2^+^Ki67^+^ double-positive cells. Overlapping expression shown in high magnification panels on the right. Scale bars, 50 μm. (**G**) Quantification of number of PAX6^+^, PAX6^+^Ki67^+^, Tbr2^+^, and Tbr2^+^Ki67^+^ cells per square millimeter in anterior coronal sections. Graphs are represented as means with SEM; *n* = 4. Unpaired *t* test; *P < 0.05.

Abnormal VZ, SVZ, and IZ development can induce or limit gyrification (*66*). We therefore examined these zones in the MCP-treated animals. Our rosettes showed MCP and other Gal-3 blockers disrupting the ABP of ZO-1. We assessed this in vivo with immunohistochemistry for ZO-1 in the pallial VZ. Control embryos had a very distinct expression of apical VZ ZO-1 expression (Fig. 7A and fig. S7B). In contrast, MCP-exposed embryos had broad ZO-1 expression, suggesting TJ disruption and expansion of the apical VZ (Fig. 7, A and B). This effect was most pronounced anteriorly and gradually diminished toward the middle and posterior portions of the pallium (Fig. 7, A and B; and fig. S7, A to E).

ZO-1 regulates the cytoskeleton and is critical for the organization of the spindle pole during cell divisions (*67*). Horizontal cell divisions have cleavage planes with ~90^o^ orientation with respect to the lateral ventricle, and this angle becomes more parallel to the LV in vertical divisions (Fig. 7C). To determine whether the preponderance of horizontal versus vertical divisions changes in MCP-exposed embryos, we measured the cleavage plane angle of dividing cells in the VZ of the neocortex. Angles below 60% were categorized as vertical divisions. Notably, the MCP-exposed embryos had greater than four times more vertical divisions compared to controls (Fig. 7, D and E).

Previous studies have shown that oblique cell divisions in the VZ lead to generation of basal progenitors (oRGs), a population of progenitors enriched in gyrencephalic species. We predicted that altered cleavage planes would cause abnormal numbers of basal progenitors (or oRGs) in mouse. These cells can be detected as Pax6^+^ proliferative progenitors in the IZ (*9*, *48*). We found significantly more Pax6^+^ progenitor cells in the IZ of embryos exposed to MCP, at all anterior posterior levels (Fig. 7, F and G; and fig. S7, F to I). We also found more Pax6^+^Ki67^+^ cells in the IZ at all anterior posterior levels in MCP-exposed embryos (Fig. 7, F and G; and fig. S7, F to I). We also show that the number of Tbr2^+^ and Tbr2^+^Ki67^+^ cells was not significantly different between controls and MCP exposed embryos (Fig. 7, F and G), indicating that the pool of intermediate progenitors was not affected. Together, these data suggest that Gal-3 blockade causes an increase in the pool of basal progenitors, as indicated by proliferative Pax6^+^ cells in the IZ, a phenotype also seen in Gal-3 binding protein (GAL3BP) mutants (*66*).

## DISCUSSION

**Fig. 8.**
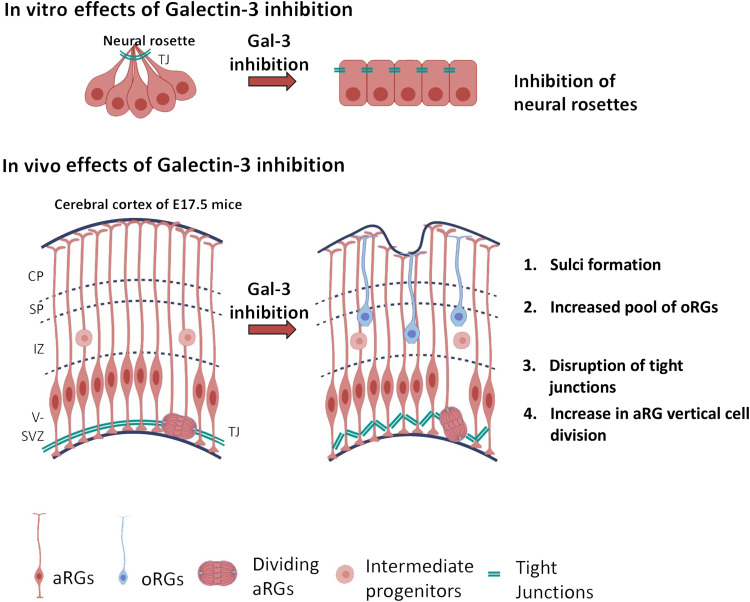
The effects of Gal-3 inhibition on ABP in neural embryonic development. Blocking Gal-3 in vitro resulted in disruption of the polarization of the neural epithelium, as evidenced by a decrease in the number of neural rosettes. In vivo, disruption of ZO1 could be observed around the VZ of E17.5 mouse embryos upon Gal-3 inhibition. The number of vertical cell divisions increased, and more Pax6^+^Ki67^+^ cells were found in the intermediate zone, indicating delamination of oRG. Last, sulci were observed in MCP and Gal-3 KO mice. TJ, tight junctions; CP, cortical plate; SP, subplate; IZ, intermediate zone; V-SVZ, ventricular-subventricular zone; aRGs, apical radial glia; oRG, outer radial glial.

We have shown that Gal-3 l.o.f. caused ABP disruption in vitro and in vivo, and a summary of our data is shown in Fig. 8. Additionally, Gal-3 KO and the Gal-3 blocker MCP during embryonic development caused sulci to appear in the murine cerebral cortex. These findings suggest that Gal-3 has fundamental roles in early mammalian brain development. MCP is readily available over the counter as a nutraceutical without health warnings. However, blocking Gal-3 function may inhibit ABP and thus be harmful for the developing fetus in pregnant women. Thus, our work cautions against the use of MCP as medication during pregnancy.

### Gal-3 regulates ABP in hESC rosette formation

Using neural induction of hESCs in vitro, we observed a peak of Gal-3 expression at day 2, preceding important early neural commitment events, such as ABP and Pax6 expression. Gal-3 KD did not alter expression of Pax6, suggesting that Gal-3 works in a Pax6-independent fashion. Gal-3 l.o.f. experiments did show, however, a decrease in the number of neural rosettes in culture, suggesting a role in cellular events leading to ABP.

Gal-3 was previously shown in other models to affect epithelialization and apical-basal compartmentalization, targeting protein cargos to the apical side (*25*, *26*). We observed Gal-3^+^ intracellular puncta in hESC-derived NSC. We confirmed that the Gal-3 puncta were colocalized with vesicles and enriched in the Rab11^+^ compartment, which labels recycling endosomes. Secreted Gal-3 can form lattices in the cell membrane upon its interaction with transmembrane receptors, which can result in incorporation into carrier vesicles (*68*–*70*). We found that Gal-3 secreted via exosomes, with a higher percentage in the early days of neural differentiation. It is also possible that Gal-3 participates in cell polarization via interaction with the extracellular domain of junctional proteins, as described in previous work (*71*–*73*). Gal-3 has also been implicated in ciliogenesis and is associated with centrosomal abnormalities (*28*). Here, we observed that junctional, cytoskeletal, and ciliary proteins, such as ZO-1, Par3, N-cadherin, F-actin, and ARL13-B, lack typical ABP organization upon Gal-3 blockade, although no direct interaction between those components was analyzed.

These findings suggested that Gal-3 could affect ABP in a number of divergent cellular and extracellular domains. This was confirmed by the variety of tools we used and the location of Gal-3 expression that they target. Gal-3 siRNA would initially have reduced cytoplasmic and nuclear Gal-3. The function blocking Gal-3 antibody and MCP would have limited Gal-3 incorporation, binding Gal-3 extracellularly and blocking its function. Recombinant Gal-3, on the other hand, would have, at first, increased the extracellular concentration of Gal-3 and then affected intracellular compartments. Thus, over time, it is likely that location of Gal-3 gain of function or l.o.f. would have spread into other domains.

### RNA-seq reveals potential mechanisms influencing Gal-3 function in ABP

The function of Gal-3 in healthy mice and in disease models in the postnatal and adult SVZ has been extensively studied (*18*, *39*–*43*, *45*, *74*). Early studies revealed that Gal-3 helps maintain V-SVZ neuroblast migration in adult mice (*18*, *40*), and, later, we showed that it regulates cell fate choices in postnatal mice via Wnt and BMP signaling (*43*, *45*). ECs have apically localized motile cilia, and these were stunted and fewer in number in Gal-3^−/−^ mice, suggesting abnormal apical pole maintenance (*18*). However, not much is known about pathways regulated by Gal-3 in the embryonic brain.

Our transcriptomics GO analysis data revealed that first Notch at day 3 and then Wnt and BMP pathways at day 6 are up-regulated upon treatment with the Gal-3 inhibitor MCP. The regulation of BMP is supported by the previous literature showing involvement of Gal-3 in these pathways (*43*). Our data also support previous literature showing that Gal-3 regulates Notch signaling, via down-regulation of JAG1 and DLL4, including via binding to JAG1 (*75*, *76*). KEGG analysis additionally showed Hippo pathway up-regulated upon MCP treatment. The Hippo pathway is not only important for development and neural differentiation but also essential for cell-cell signaling (*77*, *78*). Its main effector YAP is not only present at the junctions, but loss of junctional YAP led to loss of N-cadherin in ECs and of ZO-1 and β-catenin from cellular junctions in RGs in the developing mouse brain (*59*, *79*). We also found that Yap expression becomes disorganized in response to Gal-3 l.o.f. and shifts from TJs to the nucleus. YAP nuclear localization led to the loss of apical surface integrity, hydrocephalus, and gyrification in mice (*60*). Whereas Gal-3 expression clearly affects Yap localization, we are uncertain whether YAP is necessary for Gal-3 function. Last, we also saw dysregulation of several cadherin genes upon MCP treatment. Gal-3 has been shown to interact with cadherins (*71*).

### Gal-3 is expressed in embryonic NSC niches

As predicted by the hESC findings, we found robust Gal-3 expression in the developing human brain. Gal-3 was located in the proliferative layers, VZ, SVZ, and also in the IZ. These data suggested that it was positioned to have effects on the human stem cell niche.

In the mouse, RT-qPCR revealed *Lgals3* across different developmental stages and at different regions of the brain, with the lowest expression in the cerebral cortex. Gal-3 expression was also examined via immunostaining in E17.5 mouse embryos. Unexpectedly, Gal-3 immunoreactivity was in the subpallium and Gal-3 could not be detected in the pallium. We predict that more sensitive immunohistochemical techniques such as enzymatic amplification would detect Gal-3 in the pallium. We found strong Gal-3^+^ expression apically in VZ cells and coexpression with the stem cell marker Sox2.

### Loss of Gal-3 function generated sulci in the mouse cortex and disrupted embryonic ABP

In examining Gal-3 KO and MCP-treated brains, we noticed that approximately one-third to half of the embryos (respectively) had sulci in the cerebral cortex. A similar phenotype has been observed after overexpression of mutant versions of the human GAL3BP (*LGALS3BP*) by the Cappello group (*66*). They suggested that the phenotype resulted from a disruption of apical junctions and increased delamination of NSCs from the VZ (*66*). How Gal-3 and Gal3BP functionally interact in the developing human nervous system is poorly understood. Notably, mice do not express *LGALS3BP*; thus, our in vivo findings cannot be explained by Gal-3 interactions with the GAL3BP.

We suspected that the de novo sulci were correlated with disrupted ABP in the germinal zones and showed that Gal-3 inhibition by MCP disrupted ZO-1^+^ apical junctions. ZO-1 is expressed apically in a linear and tight pattern in VZ cells in controls. However, MCP caused its expression to spread in a honeycomb pattern, similar to the in vitro spread of ZO-1 from the apical lumen of neural rosettes in response to Gal-3 blockade. Because the large majority of cells lining the VZ during embryogenesis are RG stem cells, we believe that the ZO-1 dispersion occurred in these cells. These data suggest that Gal-3 is essential for ABP in the developing cerebral cortex.

Migration may directly influence cortical folding, and we showed that Gal-3 is necessary for postnatal SVZ neuroblast migration (*18*). Gal-3 KOs exhibited slower and complex “exploratory” migration and resulted in overall decreases in olfactory bulb neurogenesis (*18*). Similarly, it may be that, in the developing cortex, Gal-3 KO or MCP blockade disrupts rates of migration form the mitotic zones into developing cortical layers. Future live imaging of slices may shed light on this interesting possibility.

We also suspected that the ABP disruption in the VZ would affect cell division and layering of progeny. We found that Gal-3 inhibition increased the percentage of VZ vertical cell divisions by fourfold. Gal-3 binds to NuMa, affecting mitotic spindle cohesion and localization in HeLa cells (*31*). Disrupted cellular junctions may also contribute to the phenotype, as the LGN/NuMA complex localizes at cellular junctions and is crucial for division angle determination (*80*). The switch to vertical divisions suggests that the VZ may generate more oRG progenitor cells. The number of Pax6^+^ and Pax6^+^Ki67^+^ cells in the IZ was increased upon MCP exposure, which was not observed for intermediate progenitors detected with Tbr2. This could have contributed to the observed cortical folding, because oRG cells have been linked with gyrus formation and the oRG population is increased in gyrencephalic species (*49*). Alternatively, cortical folding may also be due to early delamination of progenitors from the VZ, and TJ disruption has been seen before in other studies (*60*, *65*).

### Clinical concern

Proteins associated with apical polarization are essential for development and ABP disruption can lead to abnormal brain development. Junctional instability was shown to lead to disruption of radial glial cell morphology, disruption of the ventricular surface, defects in neuronal migration, and development of hydrocephalus (*10*, *81*). Blocking Gal-3 function to treat systemic inflammation may inhibit ABP and thus be harmful for the developing fetus in pregnant women (*82*–*84*). MCP is readily available over the counter as an anti-inflammatory nutraceutical yet there are no warnings that taking it during pregnancy is contra-indicated. Further studies on human mutations of the Gal-3 gene in human cohorts could help to establish causal relations.

### Limitations of the study

Our in vitro studies were conducted in hESC line H9 only, and other cell lines may vary in their response to Gal-3 l.o.f. Similarly, we used commercial medium for neural induction in which the components are not disclosed. Thus, it is unclear to what extent our in vitro results are dependent on the exact culture medium composition.

With immunohistochemistry, we did not detect Gal-3 in the pallial VZ in E17.5 embryos, possibly caused by lack of sensitivity in detection of expression and/or peak expression earlier during development.

MCP effectively blocks Gal-3 function by binding to the noncanonical site of its carbohydrate recognition domain (*82*, *83*, *85*). Several studies have administered MCP to animals in drinking water (*54*, *56*, *86*–*89*). However, it is not clear how specific MCP is to Gal-3 (*85*).

We did not investigate whether Gal-3 regulates other aspects of ABP in the brain including in blood vessels. A recent study showed that Gal-3 participates in BBB damage in a stroke model (*64*). We showed that Gal-3 is necessary for vascular endothelial growth factor–dependent angiogenesis poststroke in mice (*41*).

Loss-of-function experiments were conducted in vivo in murine models and human cell lines. The murine model should be investigated at later time points to explore whether the alterations in cortical cell numbers persist to later stages. We did not explore whether these alterations in cortical folding and cell numbers have effects on behavior.

Last, a notable paper shows that RG can assume distinct but parallel transcriptomics trajectories, all leading to a common newborn neuron in human and ferret, but not in mouse (*90*). We do not know whether the trajectories described are regulated by Gal-3 but one of them had a high level of expression of family member, LGals1 (Gal-1).

## MATERIALS AND METHODS

### Neural induction of hESC line H9

The H9 hESC line (WA09) is an XX genotype cell line originally obtained from WiCell. Cells were kept in an incubator supplied with 5% CO_2_ and cultured at 37°C. Cells were maintained under feeder-free conditions on Geltrex (Thermo Fisher Scientific) or Matrigel hESC-Qualified Matrix (Corning) in mTeSR Plus medium (STEMCELL Technology).

Pluripotent hESCs were induced to commit to the neural lineage using STEMdiff SMADi NIM (STEMCELL Technology). In brief, 80% confluent cells were washed with Dulbecco's Phosphate Buffered Saline (D-PBS) (Sigma-Aldrich) and incubated with 0.5 mM EDTA (Thermo Fisher Scientific) for 10 min at 37°C. Cells were pelleted down at 200*g* for 5 min and resuspended to single-cell solution in NIM supplemented with 10 μM Rho-associated kinase inhibitor (ROCKi) Y-27632 (Stratech Scientific). Cells were seeded in Matrigel hESC-Qualified Matrix (Corning) coated six-well plates containing glass cover slips (VWR) or μ-Slide 18 ibiTreat (Thistle Scientific) at densities between 2000 and 4000 cell/mm^2^. Medium was changed daily, and cells were collected at several time points.

### hESC transfection and treatments

To promote transient KD of Gal-3 in H9 cells, a library of siRNAs targeting Gal-3 was acquired from Dharmacon (siGal-3). For Fig. 2 (B and C) and fig. S1C, a nontargeting pool was acquired from Dharmacon and used as control [nontargeting siRNA (siNT)]. For fig. S1D, a siLuciferase acquired from Eurofins was used as control (siNT). Reverse lipid transfection was performed. Briefly, siRNAs and Lipofectamine (Thermo Fisher Scientific) were separately diluted in Opti-MEM (Gibco). Solutions were then mixed together and incubated for 15 min before being distributed in μ-Slide 18 ibiTreat or 24 well Matrigel-coated plates. Pluripotent H9 aggregates were detached into single-cell suspension in NIM and 10 μM ROCKi (Fig. 2, B and C, and fig. S1C) or mTeSR and 10 μM ROCKi (fig. S1D). In both cases, the medium was changed 48 hours later to NIM, which was renewed daily. Cells were collected at different time points.

Carrier-free hrGal-3 (R&D Systems) was diluted in D-PBS. Cells were treated with NIM containing hrGal-3 (1 μg/ml), which was changed every other day. For Gal-3 activity inhibition experiments, cells were treated every day with rat Gal-3 antibody clone M3/38 (1 μg/ml; Santa Cruz Biotechnology) or the same quantity of species-specific IgG (Santa Cruz Biotechnology) diluted in NIM. For cell treatment with MCP (ecoNugenics), MCP was diluted in NIM to a concentration of 0.25 or 0.5%, and the medium was filtered using 0.22-μm Millex-GP Filter Units (Sigma-Aldrich). Medium was changed daily.

### Bulk RNA-seq of MCP-treated hESCs

Cells were treated with MCP for 3 or 6 days as described. RNA was extracted using the RNeasy Mini Kit (QIAGEN) according to the manufacturer’s guidelines. Messenger RNA was purified from total RNA using poly-T oligo-attached magnetic beads. After fragmentation, the first-strand cDNA was synthesized using random hexamer primers, followed by the second-strand cDNA synthesis using either dTTP for non–strand-specific library or 2'-deoxyuridine, 5'-triphosphate (dUTP) for strand-specific library. For the non–strand-specific library, it was ready after end repair, A-tailing, adapter ligation, size selection, amplification, and purification. For the strand-specific library, it was ready after end repair, A-tailing, adapter ligation, size selection, amplification, and purification. The library was checked with Qubit and real-time PCR for quantification and bioanalyzer for size distribution detection. After library quality control, different libraries were pooled on the basis of the effective concentration and targeted data amount and then subjected to Illumina sequencing. Raw data (raw reads) of fastq format were first processed through fastp software. In this step, clean data (clean reads) were obtained by removing reads containing adapter, reads containing ploy-N, and low quality reads from raw data. At the same time, Q20, Q30, and guanine-cytosine content (GC) of the clean data were calculated.

All the downstream analyses were based on the clean data with high quality. Reference genome and gene model annotation files were downloaded from genome website. We used HISAT2 (2.2.1) to build the index of the reference genome and use HISAT2 to align paired-end clean reads to the reference genome. The mapped reads of each sample were assembled by StringTie (v2.2.3) in a reference-based approach. featureCounts (2.0.6) was used to count the reads numbers mapped to each gene. Then, FPKM of each gene was calculated on the basis of the length of the gene and reads count mapped to this gene. Differential expression analysis for two conditions/groups was performed using the DESeq2 R package (1.42.0). The resulting *P* value is adjusted using the Benjamini and Hochberg’s methods to control the error discovery rate. The threshold of significant differential expression: FDR ≤ 0.05 and |log_2_(fold change)| ≥ 0.5. GO enrichment analysis of differentially expressed genes was implemented. GO terms with FDR less than 0.05 were considered significantly enriched by differential expressed genes. KEGG analysis was performed using the website https://maayanlab.cloud/Enrichr/.

### Immunocytochemistry

Cells were fixed with 4% paraformaldehyde (PFA) in phosphate-buffered saline (PBS; Thermo Fisher Scientific) for 15 min and permeabilized with 0.5% Triton X-100 (Thermo Fisher Scientific) for 10 min. The cells were blocked for 1.5 hours in blocking solution [5% bovine serum albumin (BSA) (Thermo Fisher Scientific) and 0.2% Triton X-100 in 0.1 M PBS]. After that, the cells were incubated with the primary antibodies that were diluted in antibody solution (1% BSA and 0.2% Triton X-100 in PBS) overnight at 4°C. Next day, a three times 0.1 M PBS wash of 10 min each was performed before and after incubation with secondary antibody. For this, cells were incubated at room temperature for 1.5 hours with secondary antibodies (1:500) and DAPI (Thermo Fisher Scientific; 1:1000) diluted in antibody solution. Secondary antibodies were species specific and conjugate with Alexa Fluor 488, 568, or 647 fluorophores (Invitrogen). For a complete list of antibodies used, see table S1.

### EV isolation and characterization

Conditioned medium (72 hours) was obtained from cells at pluripotency (day 0) and during neural induction (days 2 to 5 and days 5 to 7). EVs were purified using qEV Gen 2 size exclusion columns (IZON). The particle concentration of the isolated EVs was then measured using a flow NanoAnalyzer (NanoFCM). Super-resolution imaging was performed with an ONI NanoImager using the EV Profiler v2 kit (ONI), following the manufacturer’s instructions. Triple antibody staining was carried out using the tetraspanin trio detection reagent (568 nm) and the pan-EV detection reagent (488 nm) provided in the kit, along with an Alexa Fluor 647–conjugated Gal-3 antibody (BD Biosciences). The EV concentration for staining was adjusted to 1 × 10^10^ particles/ml for each imaging channel.

Imaging data acquired were analyzed using CODI (Collaborative Discovery platform from ONI, https://oni.bio/nanoimager/software/codi-software/). The “EV Cluster and Counting” analysis module was used, and all parameter settings were adjusted following the CODI user manual and were applied consistently across imaging datasets.

### Western blotting

Cells were detached, and the pellet was washed with 0.1 M PBS before lysis. Samples were then sonicated for ~10 s at an amplitude of 10% maximum in a XL-2000 Ultrasonic Liquid Processor (Misonix). Lysates were centrifuged at maximum speed (17,000*g*) for 15 min, and the supernatant containing proteins was collected. Protein content was measured using the Pierce Bradford Plus Protein Assay Reagent (Thermo Fisher Scientific) in a microplate reader (BMG Labtech) using standard curves. Proteins were treated with NuPAGE LDS Sample Buffer (Thermo Fisher Scientific), with 100 mM 1,4 dithiothreitol (DTT; Roche), and denatured by boiling at 97°C for 5 min. Samples were loaded in NuPAGE with a bis-tris gradient of 4 to 12%, on a 1.5-mm Mini Protein Gel (Thermo Fisher Scientific) and separated via electrophoresis. Proteins were then transferred to an Immobilon-FL polyvinylidene difluoride membrane of pore size of 0.45 μm (Merck Millipore). Membranes were blocked in 5% milk in PBS-Tween (PBS-T: 0.1 M PBS and 0.1% of Tween 20; Sigma-Aldrich) for 1 hour at room temperature before overnight incubation with primary antibodies in 5% milk at 4°C. Horseradish peroxidase (HRP)–labeled secondary antibodies were diluted to 1:2500 in 5% milk, and membranes were incubated for 1 hour at room temperature. Protein bands were obtained in x-ray film (Fujifilm) and developed in ECOMAX X-ray Processor (Raytech Diagnostics) or in Chemidoc XRS Gel Imaging System using Image Lab Software (Bio-Rad) after 1 min incubation with Pierce ECL Western Blotting Substrate (Thermo Fisher Scientific).

Western blotting densitometry quantification of bands was obtained in ImageJ software and is given in arbitrary units. Gal-3 levels in Fig. 1C and fig. S1C were normalized to the loading control glyceraldehyde-3-phosphate dehydrogenase. For Fig. 1C, values are relative to basal levels during pluripotency, which were the average of hESC expression. For fig. S1C, values are relative to control. Three different cell passages of pluripotent hESCs and four different neural inductions collected between days 1 and 6 were used for comparison. Ordinary one-way analysis of variance (ANOVA) followed by Dunnett’s multiple comparisons test was used for statistical analysis. Gal-3 and Pax6 levels in fig. S1D were normalized to the loading control H3, and aberrant replicates were removed. Four different neural inductions collected between days 1 and 7 were used for comparison. Mixed-effects model followed by Šídák’s multiple comparisons test was used for statistical analysis. For a complete list of antibodies used, see table S1.

### Mice

All animal experiments were approved by a local ethical review committee and conducted under the UK Animals (Scientific Procedures) Act, 1986 (ASPA), under valid personal and project licenses. Animals were bred and housed in individually ventilated cages on a 12-hour light/dark cycle in the Biomedical Sciences Building, Oxford. Water and food were given ad libitum. Cages were environmentally enriched with an activity wheel and nesting material. Wild-type (WT) C57BL/6J mice were acquired from Charles Rivers or from the University of Oxford Biomedical Services Specific Pathogens Free Breeding Unit. Gal-3 KO mice (Lgals3^tm1Ftl^) were donated by A. Mead. At different embryonic stages, postnatal and adult animals were used. For embryonic studies where timed mating was required, breeding pairs were separated when females were plugged, which was considered E0.5.

### MCP treatment in vivo

Timed matings were set up by pairing C57BL/6J WT mice. Breeding pairs were separated on plug day E0.5. On the next day, single caged females were treated with 1% MCP diluted in water. Water containing the treatment was changed every other day for 17 days and also used to prepare mash. Females were tested for dehydration via pinch test, and no differences from control animals were found. For one female of each group, weight gain was calculated, and water consumption was measured via water bottle weight, with no statistically significant differences between them. The litters from two control and two MCP-treated females were collected at E17.5. Between both litters, there were a total of 15 control embryos and 14 MCP-treated embryos.

### RT-PCR of developmental brain sections

Tissue from nine brain regions of C57BL/6J mice at different developmental stages were dissected, and RNA was extracted using the RNeasy Mini Kit (QIAGEN). Embryonic and postnatal tissue was pooled from pups within the same litter, while the adult age was from one mouse. RNA integrity was tested using the Agilent RNA Nano Kit with RNA integrity number (RIN) requirement of 8.0. One sample (one replicate of midbrain at P10) had RIN of 5.5 but had consistent results with other replicates; therefore, it was included in the final analysis. Reverse transcription was performed using the QuantiTect Reverse Transcription Kit (QIAGEN). RT-PCR was performed on an Applied Biosystems 7900HT Fast Real Time PCR System (Thermo Fisher Scientific) using QuantiTect Primer Assay (QIAGEN) and QuantiFast SYBR Green PCR Master Mix (Thermo Fisher Scientific), according to the manufacturer’s instructions.

Data were initially analyzed using SDS Software V2.4 (Thermo Fisher Scientific), and aberrant replicates were removed. Tfrc was used as housekeeping gene, and it had efficiency between 90 and 110% and coefficient of determination (*R*^2^) ≥ 0.998. Lgals3 had a minimum efficiency of 82.60% and *R*^2^ ≥ 0.993. Ordinary one-way ANOVA followed by Dunnett’s multiple comparisons test was used for statistical analysis.

### Immunohistochemistry of mouse brain tissue

Embryos were collected at E17.5, and the brains were dissected and immersed in 4% PFA for 24 hours at 4°C. All brains were then cryoprotected in 30% sucrose until they sank (24 to 48 hours) and frozen down in dry ice. A mold was used for Tissue-Tek O.C.T. (Sakura) embedding before freezing. Coronal sections of 30- to 50-μm thickness were obtained using a Leica SM2000 R sliding microtome and kept in cryoprotectant solution at −20°C.

Free-floating brain sections were washed three times in 0.1 M PBS before and after a 15-min incubation with 50 mM glycine in 0.1 M PBS to bind free aldehydes and reduce background staining. Porous well inserts were used to facilitate the transfer of the sections into different solutions. Sections were blocked in PBS^+^ [10% donkey serum (Sigma-Aldrich) and 0.5% Triton X-100 in 0.1 M PBS] for 1 hour at room temperature, followed by incubation in primary antibodies diluted in PBS^+^ at 4°C overnight. The next day, sections were washed with 0.1 M PBS before and after incubation with secondary antibodies (1:500) and DAPI (1:1000) in PBS^+^. All washes and incubations were performed on a rocker at 10 to 20 oscillations/min. Sections were mounted in FluorSave Reagent (Merck Millipore). For a complete list of antibodies used, see table S1.

### Human embryonic brain tissue immunofluorescence

One 17-PCW human fetal tissue sample from a terminated pregnancy was provided by the joint MRC/Wellcome Trust-funded Human Developmental Biology Resource, University of Newcastle. The tissue was collected with appropriate maternal consent and approval from the Newcastle and North Tyneside National Health Service (NHS) Health Authority Joint Ethics Committee Specimens. The sample was shipped under a Material Transfer Agreement (MTA H-20068526) between the University of Newcastle and the University of Oxford as part of other studies conducted in the laboratory of Professor Molnár. The donated tissue was screened for gross brain abnormalities or common genetic defects and has been found to be suitable for this study by the personnel of the tissue bank before inclusion in the study. Sex and ethnicity of the donated specimen have not been disclosed as not considered relevant for the study. After collection, the brain sample was immediately fixed in 4% PFA (in 0.1 M PBS) for at least 24 hours with minimal postmortem delay, and it was then stored in 0.1 M PBS with 0.05% sodium azide at 4°C upon arrival to avoid bacterial contamination.

The human brain sample was manually cut into coronal slabs (~0.5 to 1 cm in thickness) before embedding in 3% low–gelling temperature Agarose (Sigma-Aldrich) and cut into 100-μm-thick coronal sections using a Vibratome VT1000S (Leica). Selected free-floating sections from a medial slab containing the prospective parietal cortex were subjected to antigen retrieval by incubation in 10 mM sodium citrate in 0.1 M PBS at 65°C for 20 min. Sections were then blocked in 20% normal donkey or goat serum (Sigma-Aldrich) 0.3% Triton X-100 (blocking solution) for at least 6 hours at room temperature. The tissue was then incubated with the primary antibodies diluted in blocking solution 48 hours at 4°C. Sections were washed for 1 hour with 0.1 M PBS before incubation with the appropriate secondary antibodies diluted in blocking solution (1:500). Sections were then washed for 1 hour in 0.1 M PBS, counterstained with DAPI in 0.1 M PBS (1:1000) for 30 min at room temperature, and lastly mounted on glass coverslips with FluorSave reagent (Merk Millipore) and sealed with nail polish to avoid drying.

Due to the thickness and irregularity of the human tissue, *z*-stacks were acquired depending on the thickness of the slice, with ~1.0- to 3.0-μm thickness at 63× objective in the LEICA TCS SP5 Broadband Confocal Laser Scanning Microscope. For a complete list of antibodies used, see table S1.

### Human embryonic brain tissue immunohistochemistry

One 14-PCW human fetus from a medically induced or spontaneous abortion was fixed in Bouin’s fixative, embedded in paraffin, and cut into a series of 10-μm-thick sections in the coronal plane. The material was part of previous studies (*91*) and was donated to the laboratory of Zoltán Molnár by G. Meyer. The brain was acquired with maternal consent and approval by the Ethical Committee of the University Hospital La Laguna.

Coronal 10-μm on-slide paraffin-embedded slices were used. Paraffin was melted for 30 min at 60°C and deparaffinized in xylene. After a wash in 100% ethanol, endogenous peroxidase was blocked in 3% H_2_O_2_ in methanol for 15 min at −20°C. Sections were rehydrated by an ethanol serial concentration wash (100, 80, and 50%) for 5 min each at room temperature. The sections were washed in 1× tris-buffered saline [TBS; 10× stock solution: 0.2 M Trizma base and 1.5 M NaCl (pH7.4)] at room temperature for 20 min. For antigen retrieval, 10 mM citrate buffer solution was used to incubate the slices for 20 min at 60°C. Samples were cooled down and washed with TBS once before drying to allow circling the section with a hydrophobic pen. Unspecific antibody binding was blocked in 3% goat serum and TBS-T (TBS and 0.1% Triton X-100) for 1 hour at room temperature. Next, sections were blocked in avidin for 15 min and in biotin for 15 min to enhance the signal using the ready-to-use solution Avidin/Biotin Blocking Kit (Vector Laboratories). Sections were incubated with the primary antibody overnight at 4°C in antibody solution (1% goat serum and TBS-T). The following day, sections were washed three times in TBS-T. Sections were incubated for 3 hours at room temperature with the biotinylated secondary antibody diluted in 1:100 in antibody solution. Three 5-min washes in TBS-T and one in TBS preceded a 1-hour incubation with the VECTASTAIN Elite ABC-HRP Kit (Vector Laboratories), according to the manufacturer, to enhance the signal. A 3,3′-diaminobenzide (DAB) reaction was developed using a DAB 15 Substrate Kit, Peroxidase (HRP) (Vector Laboratories), according to the manufacturer. The reaction was blocked after 5 to 8 min by soaking slides in H2O and washing for 5 min. The sections were counterstained in hematoxylin (Sigma-Aldrich) for a few seconds and washed in running water until clear. Next, sections were dehydrated in serial dilutions of ethanol (50, 80, and 100%) for 5 min each and washed in xylene. Sections were air-dried and coverslipped using DePeX mounting medium (Serva). Slides were left to dry overnight in the flow hood and sealed with nail polish the next day.

### scRNA-seq analysis

Data analyzed here were previously generated (*61*). The t-distributed stochastic neighbor embedding (tSNE) on Weighted Gene Co-expression Network Analysis (WGCNA) plot was generated using the UCSC Cell Browser (*92*) and reannotated manually. Transcript-per-million (TPM) values for *LGASL3* expression per WGCNA cluster were exported from USCS Cell Browser. Exported TPM values were converted in TPM + 1. Six RG clusters were plotted separately (RG-early, vRG, tRG, oRG, RG-div2, and RG-div1). All other cell types were plotted together, excluding the cluster Glyc, the four unknown clusters (U1, U2, U3, and U4) and other unidentified numbers. Outliers were removed using the Robust regression and Outlier removal (ROUT) method (*Q* = 1%). *P* values were obtained from the Wilcoxon’s rank sum test.

### Image acquisition

Images were taken either at ZEISS LSM 710 or 780 Laser Scanning Confocal Microscope using ZEN software; Leica TCS SP5 Broadband Confocal Laser Scanning Microscope using Leica Application Suite Advanced Fluorescence; or Nikon ECLIPSE Ti inverted microscope system using Volocity version 7.0.0.

Mouse brain tile scans were produced using the Nikon ECLIPSE Ti inverted microscope with a 10× objective with a 10% overlap and brightness but no shading. For human brain immunohistochemistry, a Leica DMR Microscope Upright Brightfield Fluorescent and Neurolucida software (MBF Bioscience) was used. Stainings were compared to no-primary, secondary-only controls to assess nonspecific binding. Images were analyzed using ImageJ.

### Image analysis

Neural rosettes 20× images were obtained on a Nikon ECLIPSE Ti inverted microscope. Three images per well were obtained. The number of neural rosettes per image was counted manually using ImageJ and then converted to number of rosettes per square millimeter. For Fig. 2C, an average of nine wells was used for comparison. Ordinary one-way ANOVA followed by Tukey’s multiple comparisons test was used for statistical analysis.

For analysis of Gal-3 colocalization in different vesicles, 63× images with 4× or 6× zoom were obtained in the Zeiss LSM 710 confocal. Maximum projection images of five stacks at 0.5 μm of distance were obtained. A macro was created on Fiji for processing the images with Gaussian Blur for posterior removal of background and thresholding. Images of Gal-3 and vesicle markers were then converted into a binary mask, and the resulting image of the operation “imageCalculator AND” on Fiji was considered the overlap between both channels. Particles or area of the overlap channel were calculated, and the percentage over Gal-3 channel was considered “Portion of Gal-3,” while the percentage of the vesicle channel was considered “Portion of vesicle.” Average of percentage was calculated for three biological replicates, each being the average of two technical replicates.

For Fig. 2 (G′, G″, H′, and H″), an average of three wells was used for comparison. Ordinary one-way ANOVA followed by Tukey’s multiple comparisons test was used for statistical analysis. For Fig. 2 (G‴, G⁗, H‴, and H⁗), an average of three wells was used for comparison. Unpaired *t* test was used for statistical analysis.

For in vitro activated–caspase-3 analysis, images were obtained in Nikon ECLIPSE Ti inverted microscope at 20×. The number of cleaved caspase-3 (aCasp3)–positive cells and DAPI^+^ nuclei was manually counted using ImageJ, and a percentage was obtained. Three images per well were obtained. An average of three wells was used for comparison. Ordinary one-way ANOVA was used for statistical analysis.

For in vitro YAP/DAPI and YAP/ZO1 colocalization analysis, confocal images were taken at 63× with *z*-stack interval of 0.5 μm. JACoP BIOP ImageJ plugin was used to calculate the Pearson’s correlation coefficient in region of interest (nuclei: DAPI signal with Gaussian blur = 2 σ thresholded using Huang; junctions: ZO1 signal with Gaussian blur = 2 σ thresholded using Li). Automatic signal thresholding was used: Huang for DAPI, Otsu for YAP staining, and Moments for ZO1 staining. Nine images per group per repeat were analyzed. Unpaired *t* test was used for statistical analysis.

For ZO1 analysis in vivo, confocal images of the ventricular and subventricular zones of anterior, middle, and posterior sections were captured at ×40 magnification with a *z*-stack interval of 1 μm, 20 optical sections per *z* stack. The average thickness of the apical region was measured using ImageJ. Brains of six controls and six MCP-treated mice from two litters treated E17.5 mice embryos were used for this analysis. Mann-Whitney test was used for statistical analysis.

For in vivo analysis for cell division angle, RGs in anaphase located at the ventricular surface of the cortex were imaged with confocal microscope *z*-stack intervals of 0.4 μm and between 40 and 80 optical sections per *z* stack at 63× oil lens. For each embryo, 80 to 120 NSCs in anaphase were imaged. The cleavage plane angle relative to the ventricular surface was measured using ImageJ. Cleavage plane angles below 60° were classified as vertical divisions, while those at or above 60° were considered horizontal divisions. Brains of three control and three MCP-treated E17.5 mice embryos from two litters were used for this analysis. Mann-Whitney test was used for statistical analysis.

For Pax6, Tbr2, and Ki67 analysis in embryonic sections, tile scans of cortex were captured at ×63 magnification with a confocal microscope, with a *z*-stack interval of 4 μm and three optical sections per *z* stack. For each animal, anterior, middle, and posterior cortical sections measuring 250 μm in width were analyzed. The cortex was categorized into VZ and SVZ, IZ, subplate, and cortical plate on the basis of their morphological characteristics visualized through DAPI staining. Manual counting of the number of positive and double-positive cells in different cortical regions was performed using the Cell Counter tool in ImageJ. Brains of four control and four MCP-treated E17.5 embryos from two litters were used for this analysis. Unpaired *t* test was used for statistical analysis.

For quantification of cortical folding, all observed gyri and sulci were described using SD and LGI values (*65*). To calculate the SD value, a line was drawn connecting the highest point of the gyrus with the lowest point of the sulcus. The LGI was determined by the contour of the gyrus divided by the length of the surrounding gyrus. Foldings were classified as gyri/sulci if the SD value exceeded 100 μm and the GI value exceeded 0.5. Then, the percentage of animals with one or more cortical foldings in each mouse litter and overall was calculated.

To determine the GI value, the total length of the contour surrounding the brain hemisphere, including sulci, was divided by the length of brain hemisphere not including the sulci. Two brain sections per animal were used for this calculation. All animals containing sulci were quantified, and the average value was used for statistical analysis with Kruskal-Wallis test followed by Dunn’s multiple comparisons test.

For the notches analysis, a notch was defined as an indentation along the SVZ, excluding natural inflection points associated with normal ventricular curvature. All coronal brain sections in which the SVZ was visibly present were analyzed. Images were acquired at ×10 magnification, and their scale was calibrated in ImageJ. Notches measuring less than 15 μm in depth were excluded to control for artifacts introduced by histological staining. Kruskal-Wallis test followed by Dunn’s multiple comparisons test was used for statistical analysis.

### Statistical analysis

All experiments were repeated at least three times unless specified. Biological replicates were composed of different animals or different neural inductions started at different cell passages. Technical replicates were composed of different brain sections of the same animal or separated wells of same neural induction.

Analysis was performed using the statistical package GraphPad Prism 10, unless otherwise indicated. Experiments were tested for normality distribution with Shapiro-Wilk test. *P < 0.05; **P < 0.01; ***P < 0.001; ****P < 0.0001
