# Supplementary material for: Galectin-3 induces neurodevelopmental apical-basal polarity and regulates gyrification
Source: Sci Adv. 2025 Sep 3;11(36):eadt5859. doi: 10.1126/sciadv.adt5859 (PMC12407091; doi:10.1126/sciadv.adt5859)
Supplement: Supplementary file 1 — Figs. S1 to S7 Table S1 Legends for tables S2 to S4 [file sciadv.adt5859_sm.pdf]

Science Advances
AAAS

Supplementary Materials for

Galectin-3 induces neurodevelopmental apical-basal polarity and regulates gyrification

Luana Campos Soares *et al.*

Corresponding author: Luana Campos Soares, luana.campossoares@dpag.ox.ac.uk;
Francis G. Szele, francis.szele@dpag.ox.ac.uk

Sci. Adv. **11**, eadt5859 (2025)
DOI: 10.1126/sciadv.adt5859

The PDF file includes:

Figs. S1 to S7
Table S1
Legends for tables S2 to S4

Other Supplementary Material for this manuscript includes the following:

Tables S2 to S4

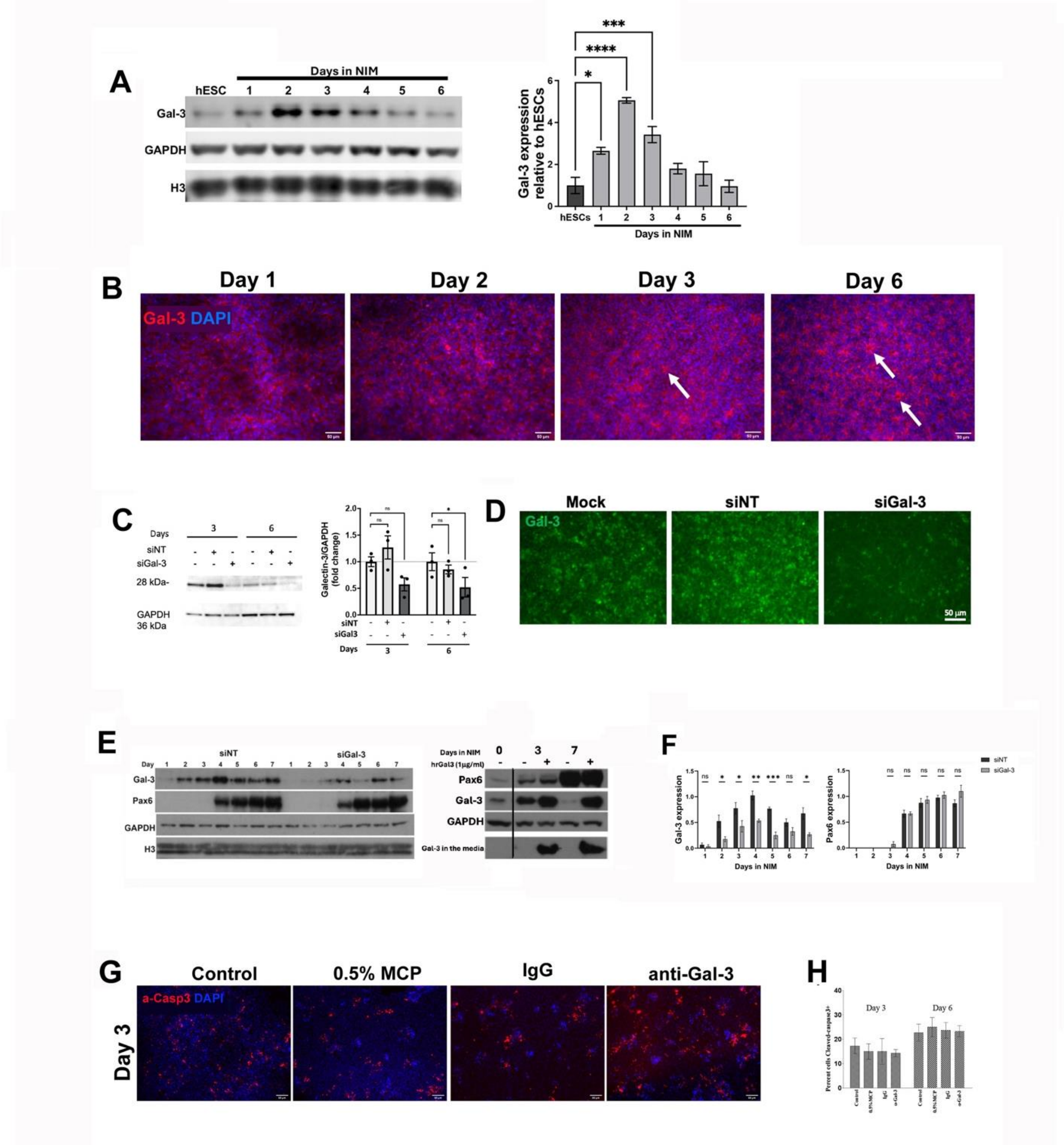

Figure S1. Gal-3 expression in neural rosettes, Gal-3 knockdown, Gal-3 expression on Pax6 and aCaspase-3.

(A) Representative Gal-3 immunoblotting of hESCs at pluripotency and at different days of neural differentiation with NIM. Quantification of Gal-3 immunoblotting normalized to GAPDH and relative to average expression at pluripotency. Shown is mean with standard error of the mean (SEM); n=3 (pluripotent hESCs) and n=4 (all Days in NIM) biological replicates. Two-way ANOVA followed by Dunnett's multiple comparison test. *$p<0.05$; ***$p<0.001$; ****$p < 0.0001$.

(B) Gal-3 immunocytochemistry after neural induction. Note the distribution of Gal-3 into apical circles visible at Days 3 and 6.

(C) Western blot showing Gal-3 siRNA knockdown in ESCs at Day 3 and Day 6. Graph is mean ±SEM, n=3 biological replicates. Fold change values were calculated relative to control. Two-way ANOVA followed by Bonferroni correction. *$p < 0.05$, ns: non-significant.
(D) Gal-3 immunofluorescence in ESC is decreased after knockdown.
(E) Western blot showing Gal-3 knockdown and treatment with hrGal3 in the media does not affect Pax6 and other markers expression in ESC.
(F) Quantification of D. Graph is mean±SEM, mixed effect model followed by Šídák's multiple comparison test. *$p < 0.05$, **$p < 0.01$, ***$p < 0.001$, ns: non-significant.
(G) Cleaved Caspase-3 immunocytochemistry at Day 3 of neural induction and treated with MCP, IgG control antibody or a-Gal-3 blocking antibody.
(H) Quantification of G shows no significant differences in the percent of cleaved caspase-3 positive cells at days 3 and 6. Graphs are mean with standard error of the mean (SEM). n= 3 wells. Two-way ANOVA.

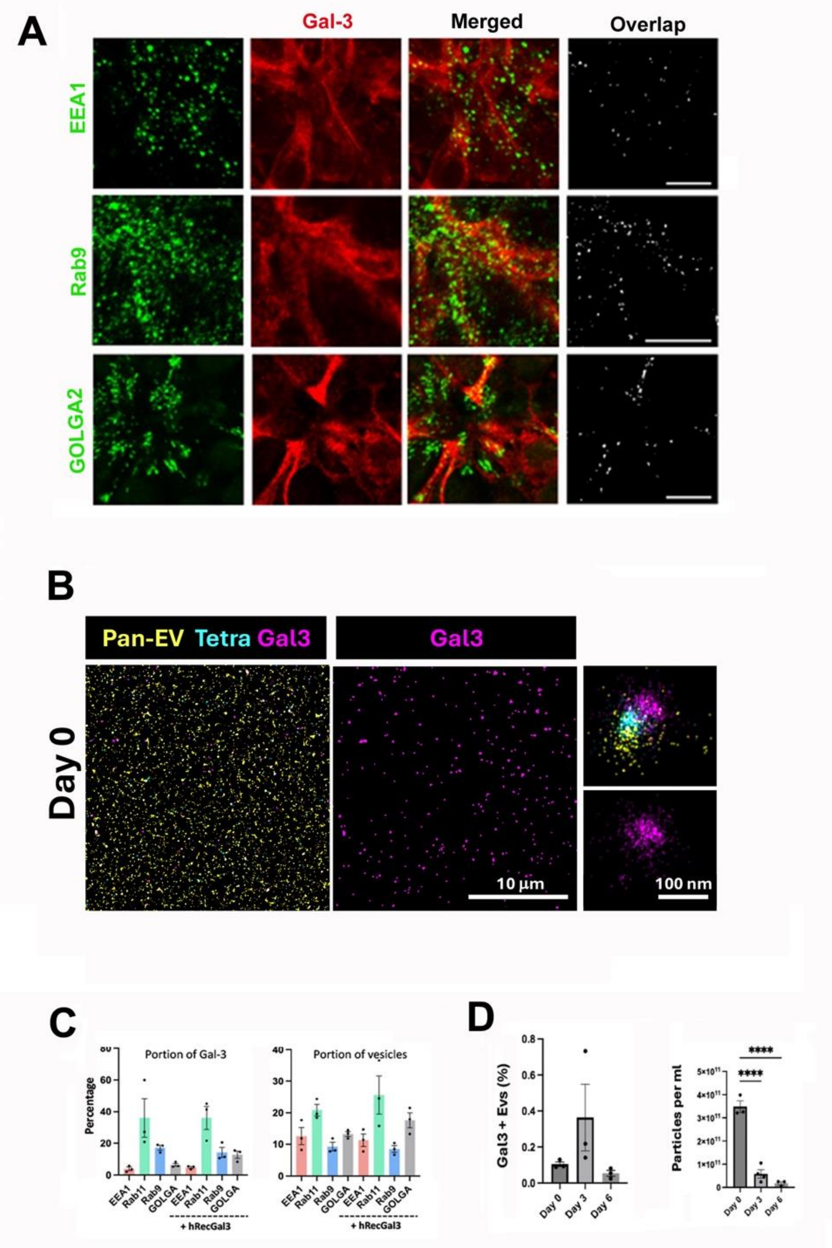

Figure S2. Gal-3 is co-localized with vesicles during neural induction of hESCs

(A) Confocal z-projection of double Immunocytochemistry of Gal-3 and EEA1, Rab9 and GOLGA2 at Day 6. Scale bar 10 µm.

(B) Representative super-resolution image of triple-positive EVs at Day 0. A small percentage of Gal-3 was co-localized with EVs (Pan-EV and Tetraspanin). Higher magnification images show the details of one single triple-positive EV. Scale bars 10 µm and 100 nm.

(C) Quantification of Manders coefficient indicating the portion of area of Gal-3 co-localized with different vesicles, and the portion of vesicles area co-localized with Gal-3 ± human recombinant Gal-3.

(D) Quantification of the percentage of Gal-3 positive vesicles at different timepoints. Quantification of number of particles per ml extracted from the conditioned media at different timepoints. Ordinary one-way ANOVA followed by Tukey's multiple comparisons test. ****$p < 0.0001$.

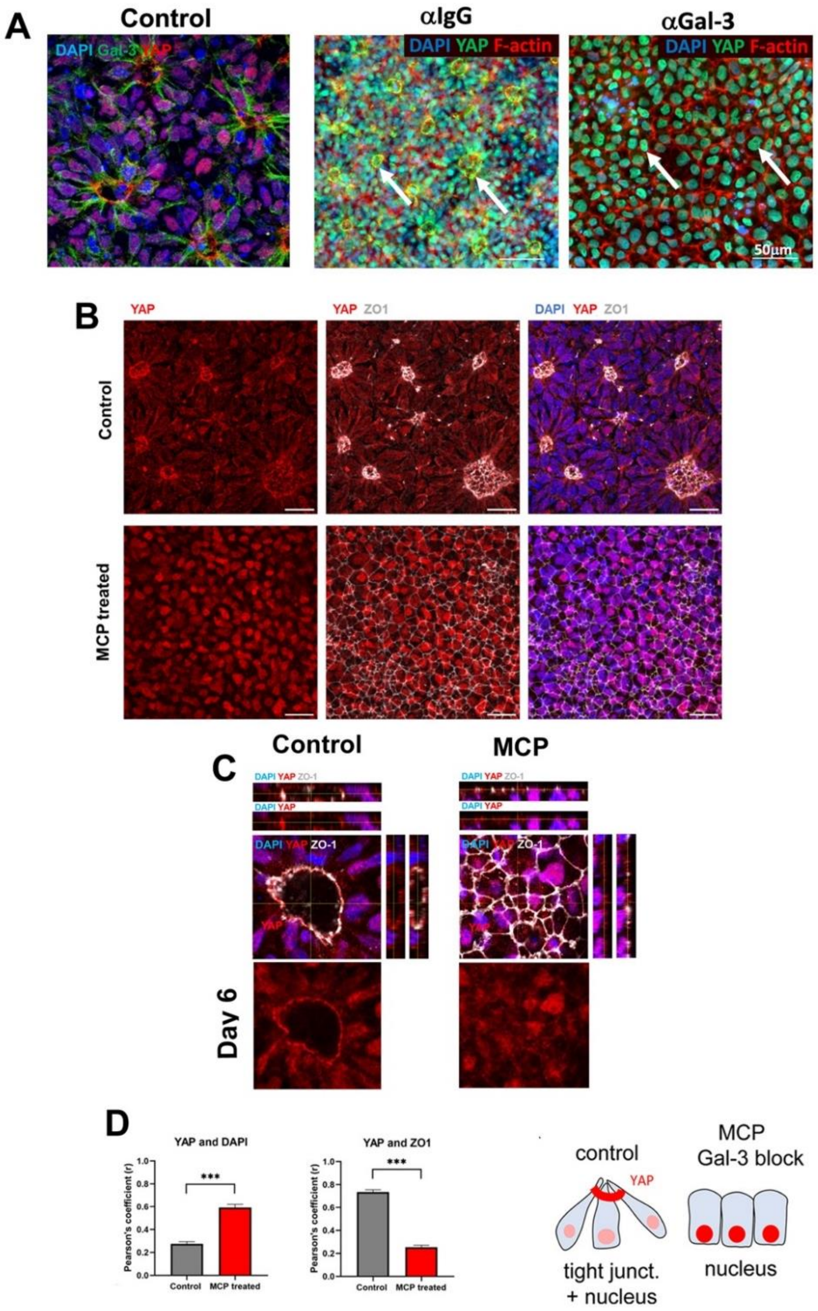

Figure S3. Gal-3 regulates distribution of and YAP recruitment to tight junctions.

(A) Immunocytochemistry showing apical Yap expression in controls and translocation to the nucleus after aGal-3 treatment. Note the increased size of the cell nucleus. Scale bar 50 µm.

(B) Representative Immunocytochemistry showing YAP and ZO-1 localization at Day 6 of neural induction in control and MCP treated cells. Scale bar 20 µm.

(C) Confocal Z stacks show that Yap and ZO1 co-localize in controls but much less so and that MCP induced YAP nuclear expression.

(D) Quantification of YAP co-localization with DAPI and YAP co-localization with ZO-1 in control and MCP treated cells at Day 6 of neural induction. Graphs are mean with standard error of the mean (SEM); n=3 biological replicates. Unpaired t test. ***p<0.001. Schematic of YAP translocation form tight junctions to the nucleus upon loss of Gal-3 function by MCP.

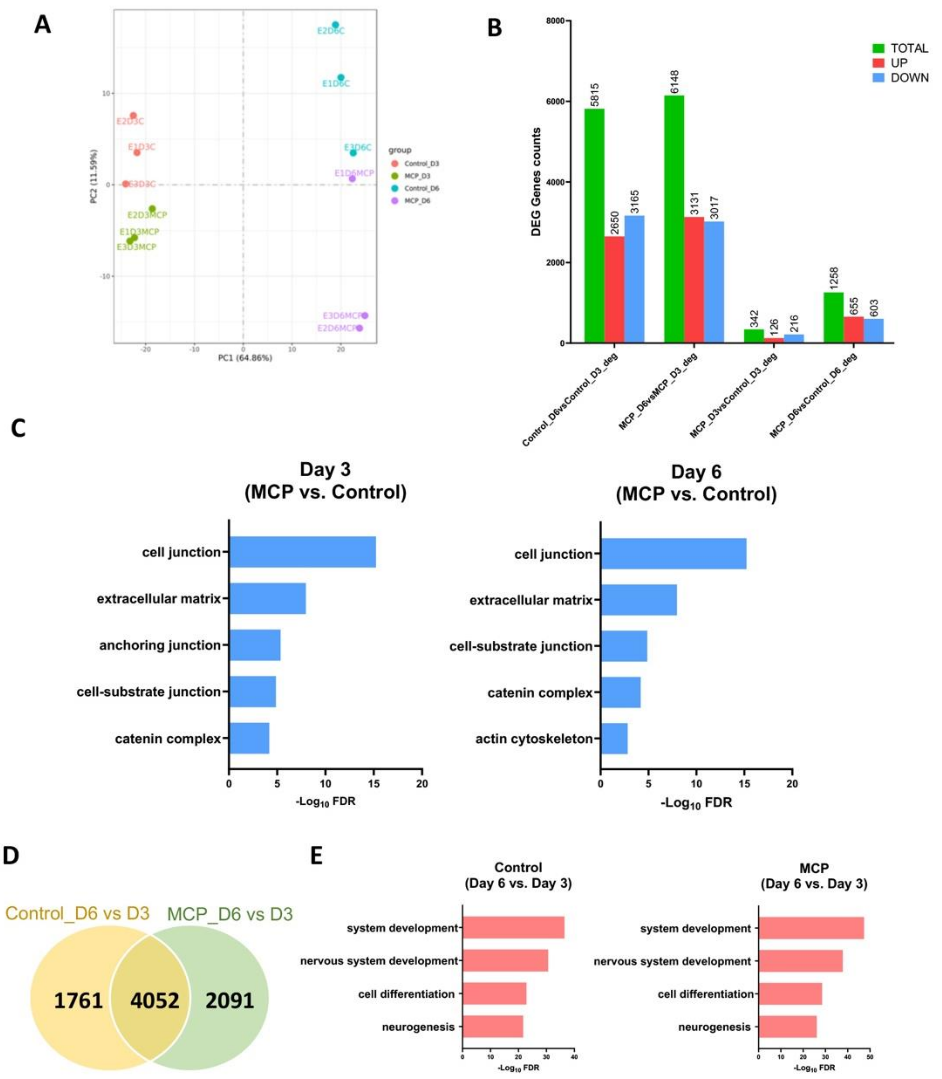

Figure S4. RNAseq reveals developmental genes regulated by Gal-3

(A) PCA analysis on the gene expression value (FPKM) of all samples showing that groups are dispersed and the sample within groups are gathered.

(B) Histogram of the number of differential genes (including up-regulation and down-regulation) for each comparison made.

(C) Gene Ontology – Cellular component enrichment analysis of genes upregulated in MCP versus control group at Day 3 and Day 6.

(D) Venn diagram representing the overlap of Differentially Expressed Genes in Day 6 vs Day 3 in Control and MCP groups. Shared genes reflect time-dependent expression independent of treatment.

(E) Gene Ontology – Biological processes of genes upregulated at Day 6 versus Day 3 in Control and MCP groups.

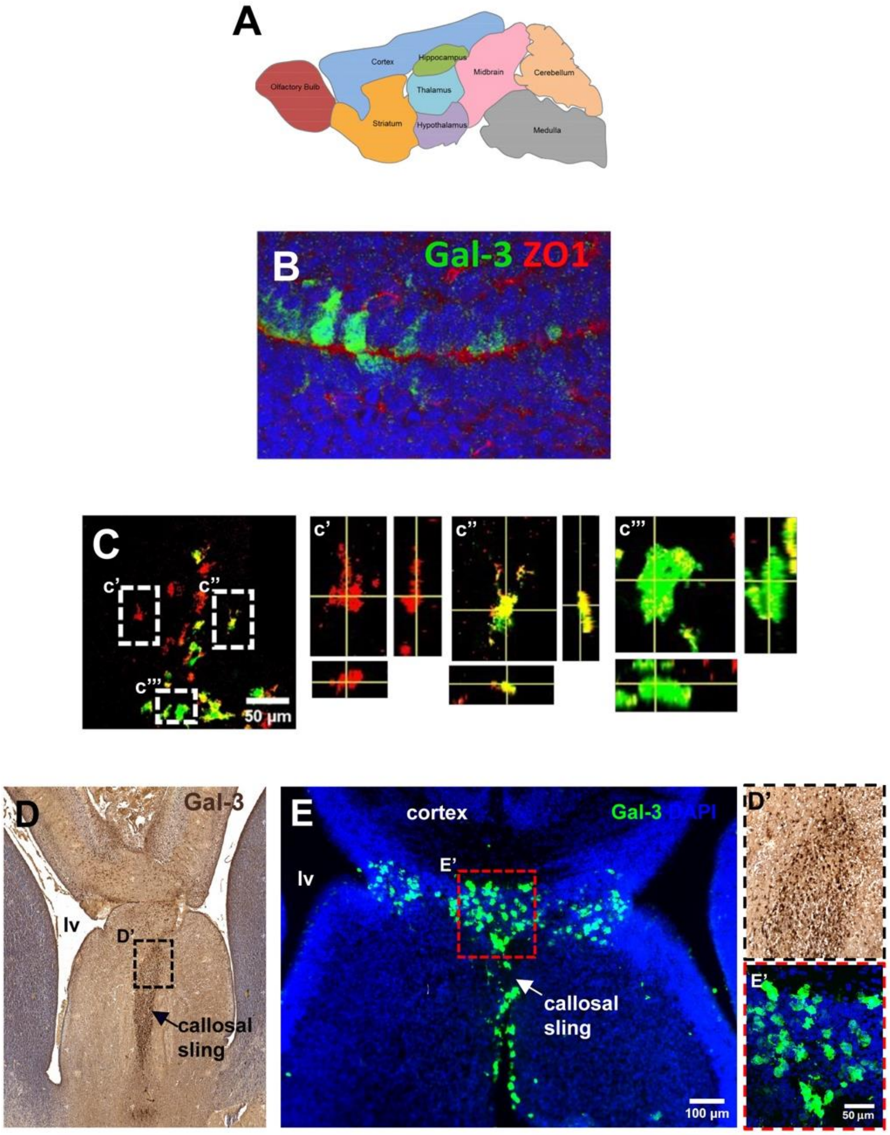

Figure S5. Gal-3 expression *in vivo*.
(A) Schematic showing areas dissected for RT-PCR.
(B) Gal-3 expression overlaps with ZO-1 expression at apical surface in an E17.5 mouse brain.
(C) 14 PCW human section showing Gal-3+ cells in the callosal sling. Scale bar 100 µm.
(D) 14 PCW human section showing Gal-3+ cells in the callosal sling. D in magnified in D'. Scale bar 100 µm.
(E) Gal-3 immunohistochemistry shows Gal-3 positive cells in the callosal sling of an E17.5 mouse. E magnified in E'. Scale bar = 100 µm.

Fig. S6

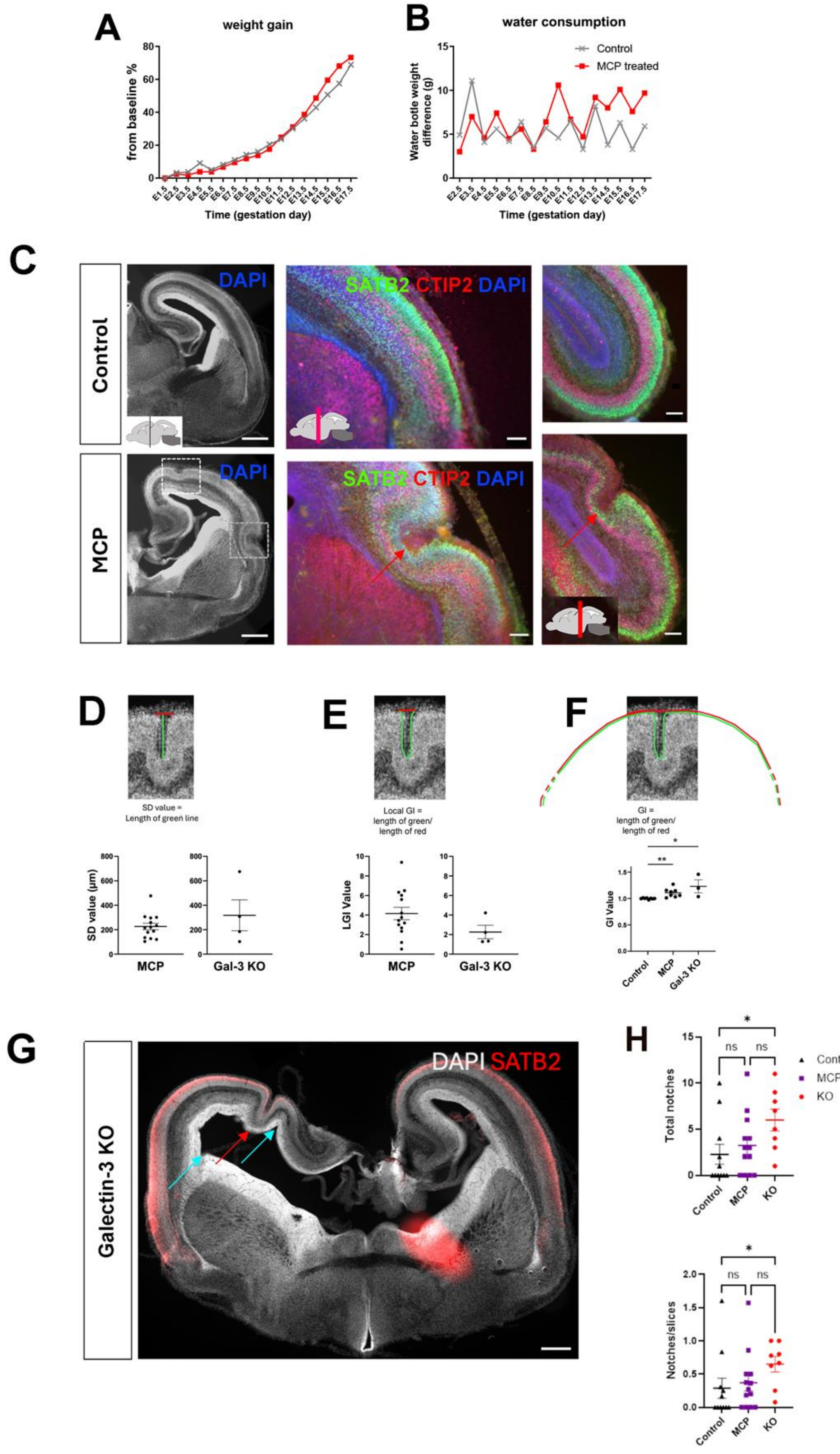

Figure S6. Gal-3 blockage *in vivo* treatment caused sulci formation.

(A) MCP administration did not affect the weight of the dams nor their water consumption (1 dam per group).

(B) Quantification of percent of embryos with 0, 1 or more sulci across litters of control and MCP treated embryos.
(C) Left panels show coronal sections of a control brain with smooth cerebral cortex and a MCP treated embryo with two sulci (red arrow). Scale bar = 500 μm. Middle and right panels show a higher magnification of the cortex stained for SATB2 and CTIP2. Scale bars = 100 μm.
(D) Photomicrographs and quantification of folds in MCP-treated embryos and Gal-3 KOs characterized using SD analysis.
(E) Photomicrographs and quantification of folds in MCP-treated embryos and Gal-3 KOs characterized using local GI analysis.
(F) Photomicrographs and quantification of GI in all embryos containing folds in the MCP-treated and Gal-3 KOs groups. Note that GI analysis encompasses the entire surface area of the cortex denoted here by red and green lines extending from the sulcus. Kruskal-Wallis test followed by Dunn's multiple comparison test. *$p < 0.05$, ** $p < 0.01$.
(G) Coronal plane-sectioned Gal-3 KO brain with DAPI+ nuclei (white) and Satb2 immunofluorescence (red). Note that the left pallium contains a prominent sulcus (red arrow) and some prominent notches in the ventricular zone (turquoise arrows). Scale bar = 500 μm.
(H) Quantification of VZ notches shows that both the total number of notches and the number of notches per section was significantly greater in the KO compared to control animals. Kruskal-Wallis test followed by Dunn's multiple comparison test. *$p < 0.05$.

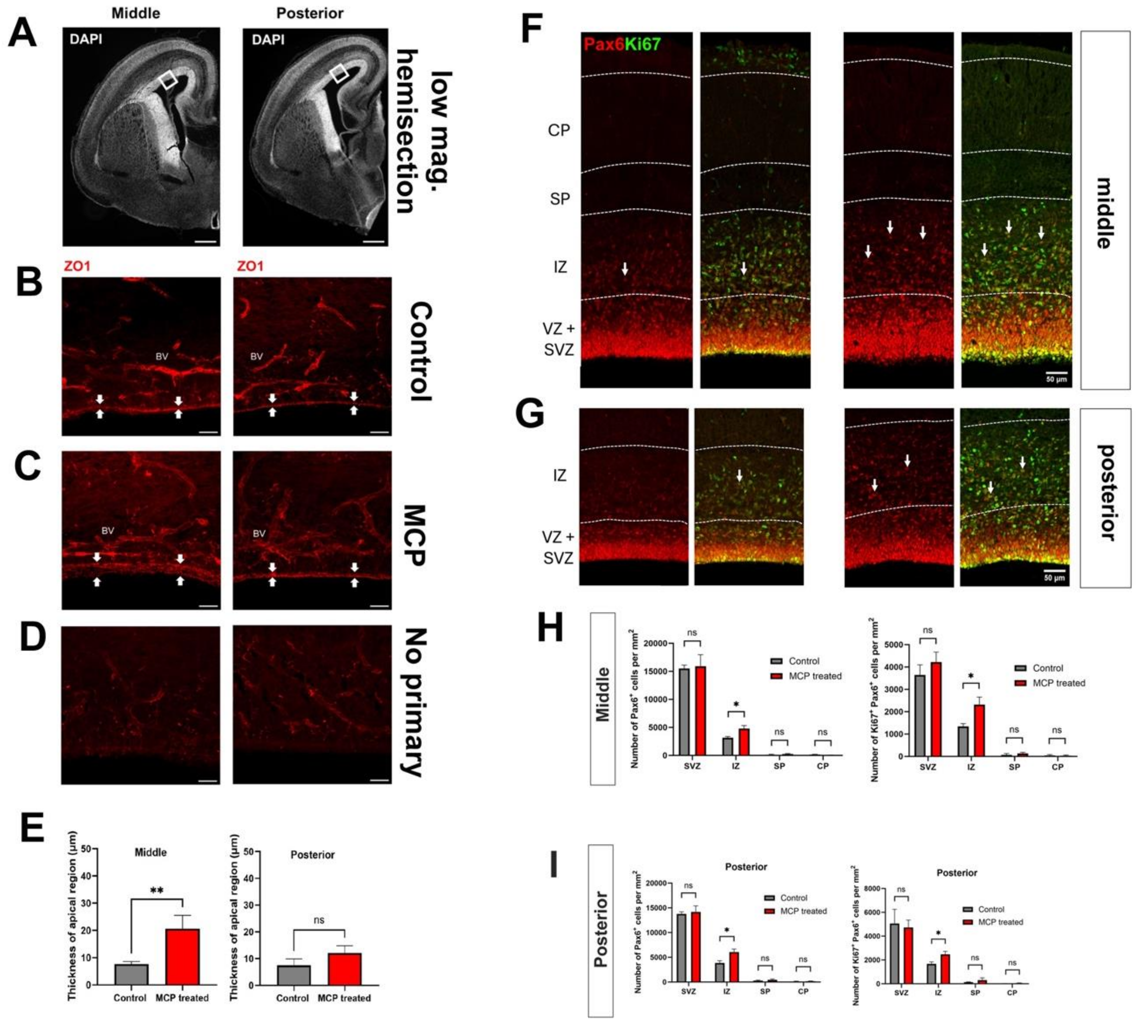

Figure S7. Effects of MCP treatment in middle and posterior sections of E17.5 embryos.
(A) DAPI stained hemi-section showing approximate area (boxes) of the pallial VZ analyzed for ABP changes. Scale bars 350 µm
(B and C) ZO-1 immunohistochemistry in control and MCP mice at middle and posterior levels. Arrows show thickness of the ZO-1 apical expression. Note that ZO-1+ blood vessels (BV) are also visible. Scale bars in C-E 50 µm
(D) No primary antibody negative control.
(E) Quantification of (B,C) showing a significantly increased area of ZO-1 expression in middle levels after MCP treatment. Mann-Whitney test $**p < 0.001$.
(F and G) Pax6 (red) and Ki67(green) immunohistochemistry in middle and posterior level sections. Note that the majority of positive cells are in the apical VZ, SVZ and IZ. Arrows show examples of double positive cells. Scale bar 50 µm.
(H) Quantification in middle and posterior sections showing significantly increased numbers of Pax6+ cells and of Pax6+Ki67+ cells in the intermediate zone. Graphs are represented as mean with SEM; n=4. Unpaired t-test. $*p < 0.05$, ns – non-significant.

Table S1.

Antibody	Source	Identifier
Rat anti-CTIP2	Abcam	Cat#ab18465<br>RRID AB_2064130
Rabbit anti-SATB2	Abcam	Cat# ab92446<br>RRID AB_2270003
Rabbit anti-Tbr2	Abcam	Cat#ab23345<br>RRID:AB_778267
Rat anti-Ki67	Invitrogen	Cat#14-5698-82<br>RRID AB_10854564
Rabbit anti-aCasp3	Cell Signaling	Cat#9661<br>RRID:AB_2341188
Goat anti-Sox2	Abcam	Cat#ab110145<br>RRID:AB_10865544
Rat IgG	Santa Cruz	Cat#sc-2026<br>RRID:AB_737202
Rat anti-Gal3	Santa Cruz	Cat#sc-23938<br>RRID:AB_627658
Rabbit anti-Gal3	Santa Cruz	Cat#sc-20157<br>RRID:AB_2136784
Alexa Fluor® 647 Rat anti-Gal3	BioLegend	Cat#125408<br>RRID:AB_1186110
Rabbit anti-Pax6	BioLegend	Cat#901301<br>RRID:AB_2749901
Rabbit anti-YAP	Cell Signaling	Cat#14074<br>RRID:AB_2650491
Rabbit anti-YAP	Cell Signaling	Cat#4912<br>RRID:AB_2218911
Mouse anti-YAP	Santa Cruz	Cat# sc-101199<br>RRID:AB_1131430
Mouse anti-ZO1	Invitrogen	Cat#33-9100<br>RRID:AB_87181
Acetylated mouse anti-Tubulin (IgG2b)	Sigma-Aldrich	Cat#T6793<br>RRID:AB_477585
Mouse anti-Gamma tubulin (IgG1)	Sigma-Aldrich	Cat#T6557<br>RRID:AB_477584
Mouse anti-N-cadherin	Cell Signaling	Cat#14215<br>RRID:AB_2798427
Rabbit anti-ARL13B	Proteintech	Cat# 17711-1-AP<br>RRID:AB_2060867
Mouse anti-ARL13B	Santa Cruz	Cat# sc-515784<br>RRID:AB_2890034
Rabbit anti-Par3 antibody	Merck Millipore	Cat#07-330<br>RRID:AB_2101325

Mouse anti-Rab11	BD Biosciences	Cat#610656<br>RRID:AB_397983
Mouse anti-Notch	Santa Cruz	Cat# 5118<br>RRID:AB_10621426
Rabbit anti-Rab9	Cell Signaling	Cat# 3288<br>RRID:AB_2096811
Rabbit anti-EEA1	Cell Signaling	Cat# 11308-1-AP<br>RRID:AB_2115327
Rabbit anti GOLGA2/GM130	Proteintech	Cat# sc-376403<br>RRID:AB_11149738
Rabbit anti-Iba1	Wako	Cat#019-19741<br>RRID:AB_839504
Mouse anti-P(S55)Vimentin	Abcam	Cat#ab22651<br>RRID:AB_447222
Goat anti-chicken Alexa Fluor 488	Molecular Probes	Cat#A11039<br>RRID:AB_142924
Goat anti-rabbit Alexa Fluor 488	ThermoFisher	Cat#A11034<br>RRID:AB_2576217
Donkey anti-rabbit Alexa Fluor 488	ThermoFisher	Cat#A21206<br>RRID:AB_2535792
Donkey anti-mouse Alexa Fluor 488	ThermoFisher	Cat#A21202<br>RRID:AB_141607
Donkey anti-rat Alexa Fluor 488	ThermoFisher	Cat#A21208<br>RRID:AB_2535794
Donkey anti-rabbit Alexa Fluor 568	ThermoFisher	Cat#A10042<br>RRID:AB_2534017
Donkey anti-mouse Alexa Fluor 568	ThermoFisher	Cat#A10037<br>RRID:AB_11180865
Goat anti-mouse IgG2b Alexa Fluor 568	ThermoFisher	Cat#A21144<br>RRID:AB_2535780
Goat anti-rat Alexa Fluor 568	ThermoFisher	Cat#A11077<br>RRID:AB_2534121
Donkey anti-rabbit Alexa Fluor 647	ThermoFisher	Cat#A-31573<br>RRID:AB_2536183
Donkey anti-mouse Alexa Fluor 674	ThermoFisher	Cat#A31571<br>RRID:AB_162542
Goat anti-mouse IgG1 Alexa Fluor 647	ThermoFisher	Cat#A21241<br>RRID:AB_2535810
Goat anti-rat Alexa Fluor 647	ThermoFisher	Cat#A21247<br>RRID:AB_141778
Chicken anti-rat Alexa Fluor 647	ThermoFisher	Cat#A21472<br>RRID:AB_2535875
Donkey anti-goat Alexa Fluor 647	ThermoFisher	Cat#A-21447<br>RRID:AB_2535864

Goat anti-Rabbit IgG - H&L Pre-adsorbed, Biotin Conjugated	Abcam	Cat#ab7089<br>RRID:AB_954829

Table of Antibodies
List of antibodies used and their sources.

Captions for Tables S2-S4

Table S2. Table of Differentially Expressed Genes
Differentially Expressed Genes (DEG) used for further enrichment analysis are highlighted [FDR <= 0.05 & |log2(foldchange)| >= 0.5]. A list of genes either UP and DOWN regulated are also shown.

Table S3. Table of enriched Gene Ontology terms
List of all significantly enriched Gene Ontology (GO) term including Cellular component (CC) and Biological Process (BP).

Table S4. Table of enriched KEGG pathways
List of significantly enriched Kyoto Encyclopedia of Genes and Genomes (KEGG) pathways in MCP treatment versus control at Day 3 and Day 6.
